# A mechanistic Individual-based Model of microbial communities

**DOI:** 10.1371/journal.pone.0181965

**Published:** 2017-08-03

**Authors:** Pahala Gedara Jayathilake, Prashant Gupta, Bowen Li, Curtis Madsen, Oluwole Oyebamiji, Rebeca González-Cabaleiro, Steve Rushton, Ben Bridgens, David Swailes, Ben Allen, A. Stephen McGough, Paolo Zuliani, Irina Dana Ofiteru, Darren Wilkinson, Jinju Chen, Tom Curtis

**Affiliations:** 1 School of Mechanical & Systems Engineering, Newcastle University, Newcastle upon Tyne, United Kingdom; 2 School of Biology, Newcastle University, Newcastle upon Tyne, United Kingdom; 3 School of Computing Science, Newcastle University, Newcastle upon Tyne, United Kingdom; 4 School of Mathematics & Statistics, Newcastle University, Newcastle upon Tyne, United Kingdom; 5 School of Chemical Engineering and Advanced Materials, Newcastle University, Newcastle upon Tyne, United Kingdom; 6 School of Civil Engineering & Geosciences, Newcastle University, Newcastle upon Tyne, United Kingdom; University of Manchester, UNITED KINGDOM

## Abstract

Accurate predictive modelling of the growth of microbial communities requires the credible representation of the interactions of biological, chemical and mechanical processes. However, although biological and chemical processes are represented in a number of Individual-based Models (IbMs) the interaction of growth and mechanics is limited. Conversely, there are mechanically sophisticated IbMs with only elementary biology and chemistry. This study focuses on addressing these limitations by developing a flexible IbM that can robustly combine the biological, chemical and physical processes that dictate the emergent properties of a wide range of bacterial communities. This IbM is developed by creating a microbiological adaptation of the open source Large-scale Atomic/Molecular Massively Parallel Simulator (LAMMPS). This innovation should provide the basis for “bottom up” prediction of the emergent behaviour of entire microbial systems. In the model presented here, bacterial growth, division, decay, mechanical contact among bacterial cells, and adhesion between the bacteria and extracellular polymeric substances are incorporated. In addition, fluid-bacteria interaction is implemented to simulate biofilm deformation and erosion. The model predicts that the surface morphology of biofilms becomes smoother with increased nutrient concentration, which agrees well with previous literature. In addition, the results show that increased shear rate results in smoother and more compact biofilms. The model can also predict shear rate dependent biofilm deformation, erosion, streamer formation and breakup.

## Introduction

Bacterial biofilms are microbial communities of single or multiple species that are attached to surfaces and encased in a self-produced extracellular matrix, which offers protection against environmental stresses such as shear forces (due to fluid flow) or bacterial invasion [1]. Mechanically stable biofilms are advantageous to environmental engineers in applications which include wastewater treatment, soil remediation and groundwater protection [2]. Conversely, biofilms can be harmful when found on medical implants or natural surfaces within the human body, where they can cause infection [3]. Biofilms also lead to bio-corrosion of pipes [4] and contamination in food processing plants, causing food spoilage [5]. For these applications it is desirable to be able to predict biofilm formation and its mechanical characteristics [6], with the aim of controlling biofilm formation and facilitating biofilm removal.

Computational biofilm models which consider spatial variation can be divided into two general classes according to the way the biomass is represented: discrete units representing individual microbes, or a continuum representing the whole biofilm. Discrete unit models such as the Cellular Automaton (CA) [7] or Individual-based Model (IbM) [8], have been developed and are now being widely applied to study biofilm formation. Models which use individuals as a basic unit have occasionally been used in ecology since 1970s, but only since the influential review of Huston et al. [9] has IbM been an explicitly delineated approach to ecological modelling. For biofilm modelling, [Kreft et al. [8],Kreft et al. [10]] initially proposed IbM as a robust tool compared with the existing CA method. In CA models, the biofilm is represented by a collection of bricks, in which each brick is represented by a Cartesian grid cell and biomass can be shifted in a finite number of directions according to a set of rules [7,11–15]. In a conventional IbM, the spatial location of each individual is not constrained by a grid. Bacterial cells or biomass agents are represented as rigid spheres, with each cell/agent having a set of variable parameters including position, volume, velocity, mass, growth rate, genotype, and so on. Each cell grows by consuming nutrients and divides when a certain volume or mass is reached. The pressure build-up due to the growth of biomass is released by maintaining a minimum distance between the neighbouring cells. Two-dimensional numerical simulations have demonstrated that IbMs produce more confluent, rounded biofilm structures than CA based models, due to the IbM allowing directionally unconstrained spreading of the biomass [16].

An open source framework which fosters collaboration is desirable in science, especially in a multi-disciplinary field such as biofilm modelling. This enables researchers to build directly upon their predecessors’ efforts, saving time, resources and increasing efficiency. Open-source software is generally free, continually evolving, and users are not “locked into” a particular strategy or company. There are several open source software packages that can simulate microbial communities such as BacSim [8], iDynoMiCS [17], and BSim [18]. The common feature of these models is that they have addressed biological processes in detail, but physical processes such as mechanical interactions and adhesion have not been modelled in detail. Among these models, iDynoMiCS has been widely used by microbial researchers for individual-based studies of microbial communities and bioreactors [19,20]. In iDynoMiCS, a representative volume is chosen from the full-scale bioreactor. The model is then scaled-up to the full reactor scale by assuming that the reactor is completely mixed and is made up of replicates of the representative volume. At present, iDynoMiCS has a limitation in that it lacks “a first principles” approach to physical interactions, whereas in reality the emergent properties of a biofilm are derived from the interaction of chemical, biological and mechanical processes. Recently, researchers have sought to incorporate biofilm mechanics in IbM [21–24]. In these models, the mechanical interactions between the particles are implemented using Hertzian or spring models. This first generation of mechanically credible models have only implemented limited biological processes and are not open-source.

The work presented here is the first step towards the development of a much needed comprehensive IbM which integrates biology, chemistry and physics to enable the prediction of the emergent properties of a wide range of bacterial communities. The model combines biological processes with mechanical interactions between individual bacteria within a community. The model has been implemented in the mechanically sophisticated Large-scale Atomic/Molecular Massively Parallel Simulator (LAMMPS), which is an open source C++ molecular dynamics (MD) code developed by Sandia National Laboratories (http://lammps.sandia.gov/). LAMMPS has been widely adopted to simulate atoms or, more generally, as a parallel particle simulator at the atomic, meso, or macro scales for a wide range of non-biological materials (e.g. metals, semiconductors, polymers) [25–27] due to its parallel, modular and extensible nature. However, it has never been employed to simulate microbial communities.

The model implemented in LAMMPS consists of two types of sub-model: the biological sub-models which handle biological processes (growth, division, decay, death), and the physical sub-models which handle physical processes (adhesion, contact, detachment). These are described in detail in the following sections. Several case studies have been explored with this novel IbM to assess its ability to predict biofilm formation, deformation and detachment when subjected to different environmental conditions.

## Method

The model is described through the well-known ODD protocol (Overview, Design concepts and Details) which is a standard protocol for simply and efficiently describing IbMs [28,29].

### Model overview

**Fig 1:**
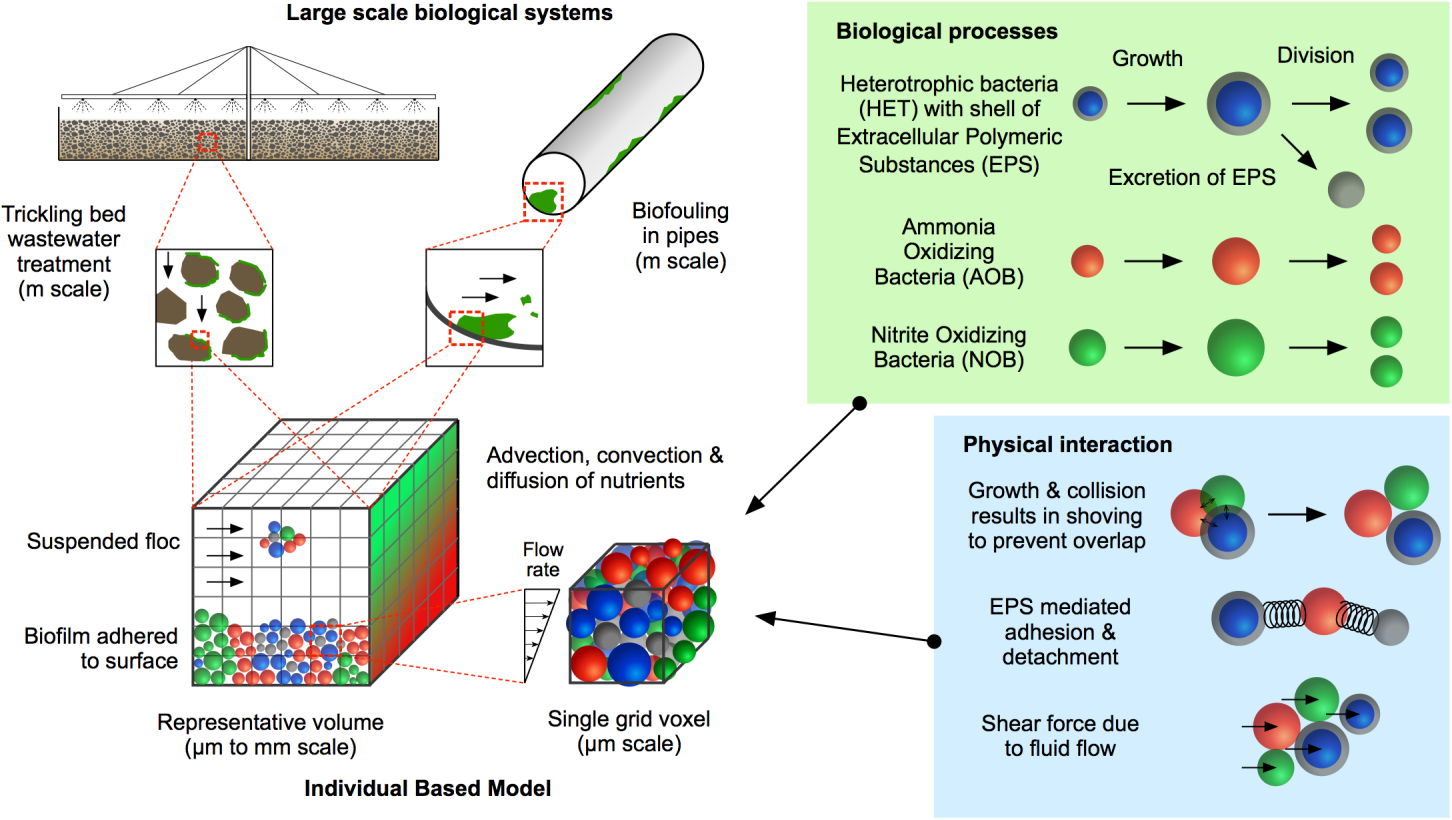
Summary of the model. A representative volume is chosen from the large-scale biological system and this representative volume is the computational domain. A generic individual based model can predict the growth and emergent properties of biofilms and flocs in a range of large-scale biological systems.

The purpose of the work is to present a generalised three-dimensional, multi-species IbM of planktonic bacterial communities, biofilms and/or flocs (a floc is a group of aggregated cells suspended in water). The model combines fundamental biological processes and physical interactions to simulate the growth of microbial communities and their response to fluid flow at the micro-scale. The model is intended to be used to predict the behaviour of microbial communities, for example activated sludge and trickling filter processes in wastewater treatments plants (Fig 1). The ultimate goal is to use the model to optimise the design of large-scale biological systems to improve their function (for example, operation of a wastewater treatment process at low temperature/pH or removal of specified pollutants).

A micro-scale rectangular volume (i.e. a cuboid) with the dimensions of *L*_*x*_×*L*_*y*_×*L*_*z*_ is chosen as the computational domain, which represents a sub-space in a macro-scale bioreactor or any other large-scale microbiological system (representative volume in Fig 1). For a completely mixed reactor, it can be assumed that the macro-scale ecosystem is made up of replicates of the micro-scale computational domain as commonly found in the literature [30,31]. However, for large-scale biological systems such as trickling bed filters or biofouling in pipes, many different sub-spaces need to be modelled to predict the behaviour of the complete system. The micro and macro scales can be coupled through appropriate boundary conditions including nutrient concentrations and flow fields. Based on the work of Ofiteru et al. [32] and Alpkvist et al. [33], three functional groups of microorganism and two inert states are considered in the model, which include active Heterotrophs (HET), Ammonia Oxidizing Bacteria (AOB), and Nitrite Oxidizing Bacteria (NOB), and their inactive counterparts, Extracellular Polymeric Substances (EPS) secreted by heterotrophs, and dead (or inert) agents (I). Each agent has four state variables: location, mass, radius, and functional group type. The computational domain is discretised into Cartesian grid elements (voxels). Each voxel has three state variables; location in the overall grid, nutrient concentrations, and fluid flow velocity.

### Design concepts

The relevant components of the design concepts of the ODD protocol are described below:

*Emergence*: the IbM is designed so that the distribution of the agents in the space, and their properties, emerge from local interactions due to growth, division, decay and physical interaction.

*Sensing*: in this model, each agent senses the nutrient concentrations within the voxel corresponding to the location of the agent’s centre. The nutrient concentrations determine the growth rate of the agent.

*Interactions*: the interactions among agents are local. The direct interactions are introduced through contact forces between the agents and EPS-mediated binding forces. The agents also interact indirectly when they share nutrient resources.

*Stochasticity*: the size and placement of daughter agents after division, and the placement of EPS particles after EPS secretion events, have been implemented in this model as stochastic processes. This enables the model to capture the stochasticity of the biological system.

*Observation*: at each time step, the state variables for agents and voxels are recorded.

### Details of the sub-models

Due to very different time scales between the physical and biological processes in this model, it is necessary to implement sub-models which operate at different time scales, and then integrate those sub-models to obtain the functionality of the entire model. The separation of time scales promotes computational efficiency. Typically, nutrient transport is very fast relative to biomass growth [12]. The mechanical relaxation occurs at a timescale between diffusion and biomass growth processes. Similar to previous work [8,12,17], diffusion is assumed to be in a quasi-steady state during biomass growth. As such, the smallest (*O*(0.0001s)) and the largest (*O*(1000 s)) time steps are used for nutrient diffusion and biomass growth, respectively. The time step used for mechanical relaxation to update the locations of agents to get the mechanical equilibrium is in between (i.e., *O*(0.01s)). The timescale of nutrient convection, fluid flow and shear of the present work is about *O*(10 s). The calculation starts with the initial nutrient concentration fields and initial bacteria inoculation. The first step is to calculate bacterial growth and division, and then update nutrient concentrations in the computational domain. At the end of this step the group of agents are mechanically unstable due to particle overlap. Thus, mechanical relaxation is performed until force equilibrium of the biofilm/floc is obtained. Force equilibrium is determined when the growth-induced residual stress (internal pressure) of the system is sufficiently relaxed, in which case the biofilm/floc shape has converged to a steady state (see the Results and Discussion section). The pseudocode of the model is given in the Supporting information (S1 File). More details about the sub-models of biological and physical processes are provided below.

#### Agent growth, decay

The growth and decay of active agents and decay of inactive agents of biomass (i.e., HET, AOB, NOB, EPS, I) are calculated using the following growth kinetic equation:

$$\frac{dm_{i}}{dt}=r_{i}m_{i}$$ (1)

Here, *m*_i_ is the mass of the particulate component and *r*_*i*_ is the specific growth/decay rate. The specific growth rate is determined by Monod kinetic equation and decay is assumed to be the first order. The specific growth/decay rates for various processes are listed in Table A in S1 File. When nutrients are lacking, the active agents shrink. If their diameters reduce to 0.8 μm, they become dead agents (I) and then decay into substrate to be consumed by other agents. The mass of decaying EPS agents is also converted to substrate in the same manner. For computational efficiency, decayed particles are removed from the simulation when they shrink down to 0.1 μm. When calculating growth and decay rates, the nutrient concentrations (*S*_S_, *S*_NH4_, *S*_NO2_, *S*_NO3_, *S*_O2_) in the expressions given in Table A in S1 File are the nutrient concentrations at the voxel where the particulate component resides. The above kinetic equation is discretized using an Euler explicit scheme with biological time step Δ*t*_*bio*_. Further details about these calculations are given in the S1 File.

#### Nutrient uptake rates

The stoichiometric matrix for particulate and soluble components is shown in Table B in S1 File. The nutrient uptake rates for each soluble component at each voxel can be calculated using Tables A and B as explained in the Supporting information (S1 File).

#### Agent division

The biomass and EPS densities are constant, thus agent diameter is calculated from the agent mass and density. If the diameter of the agent with mass (*m*) reaches a user-defined threshold (in this study, 1.25μm), it divides into two daughters. One daughter has a mass between *m*/2-10%.*m* and *m*/2+10%.*m* (a random value is drawn from a uniform distribution between these two limits) and the other takes the remaining mass to ensure that the total mass is conserved. One daughter agent takes the location of the mother agent while the centre of other daughter agent is placed in a random direction at a distance *d* (distance between the centres of both agents) corresponding to the sum of the diameters of the daughters.

#### Nutrient mass balance

Nutrient distribution within the rectangular computational domain is calculated by solving the advection-diffusion-reaction equation for each soluble component *S* (*S*_S_, *S*_NH4_, *S*_NO2_, *S*_NO3_, *S*_O2_). For given nutrient *S*, the mass balance equation is given by,

$$\frac{\partial S}{\partial t}+\vec{U}\cdot \nabla S=\nabla \cdot (D_{e}\nabla S)+R$$ (2)

The nutrient uptake rate *R* and the effective diffusion coefficient *D*_e_ are calculated based on local biomass concentrations as described in the Supporting information (S1 File). $\vec{U}$ is the fluid flow velocity. The transport equation is discretized on a Marker-And -Cell (MAC) uniform grid and the scalar *S* is defined at the centres of the voxel (cubic grid element). The temporal and spatial derivatives of the transport equation are discretized by Forward Euler and Central Finite Differences, respectively. This equation is solved for the steady state solution of the concentration fields.

#### EPS formation

Initially EPS grows as a shell around HET agents. Once the EPS shell diameter reaches 1.25 times the diameter of HET, the EPS shell is excreted as an EPS particle to the surrounding environment (the excreted EPS agent is placed next to the HET agent, but in a random direction similar to cell division). The excreted EPS particle decays to substrate. More details about modelling EPS formation is seen in [17,34].

#### Mechanical relaxation

When agents grow and divide, the system can be far from mechanical equilibrium due to agent overlap and hence mechanical relaxation is required to update the locations of the agents and minimize the stored mechanical energy of the system. Mechanical relaxation is carried out using the Discrete Element Method (DEM). In DEM, the Newtonian equation of motion of each particle is solved in a Lagrangian framework [35] which updates the location of the particle. The equation for the translational movement of particle *i* is given by:

$$m_{i}\frac{d{\vec{v}}_{i}}{dt}={\vec{F}}_{i}^{}={\vec{F}}_{c,i}+{\vec{F}}_{f,i}+{\vec{F}}_{a,i}$$ (3)

where ${\vec{v}}_{i}$ = translational velocity; *m*_*i*_ = agent mass; ${\vec{F}}_{i}$ = net force, *t* = time.

The net force acting on an agent is calculated as the sum of the contact, adhesion, and fluid forces acting on it. The contact force (${\vec{F}}_{c}$) is calculated as the sum of all the forces due to interactions between neighbouring agents. The adhesive force (${\vec{F}}_{a}$) is calculated as the sum of all pair-wise adhesive interactions due to the presence of EPS. The fluid-particle interaction force (${\vec{F}}_{f}$), or the drag force, is determined according to the drag force due to Stokes flow over a sphere. During mechanical relaxation, the neighbouring agents that are in actual contact or in close vicinity to each other are identified using the agents’ locations. Then, the total force is calculated to take account of the inter-agent viscoelastic contact forces, EPS-mediated binding forces and drag forces. Further details about the force calculations are described below.

#### Contact force

**Fig 2:**
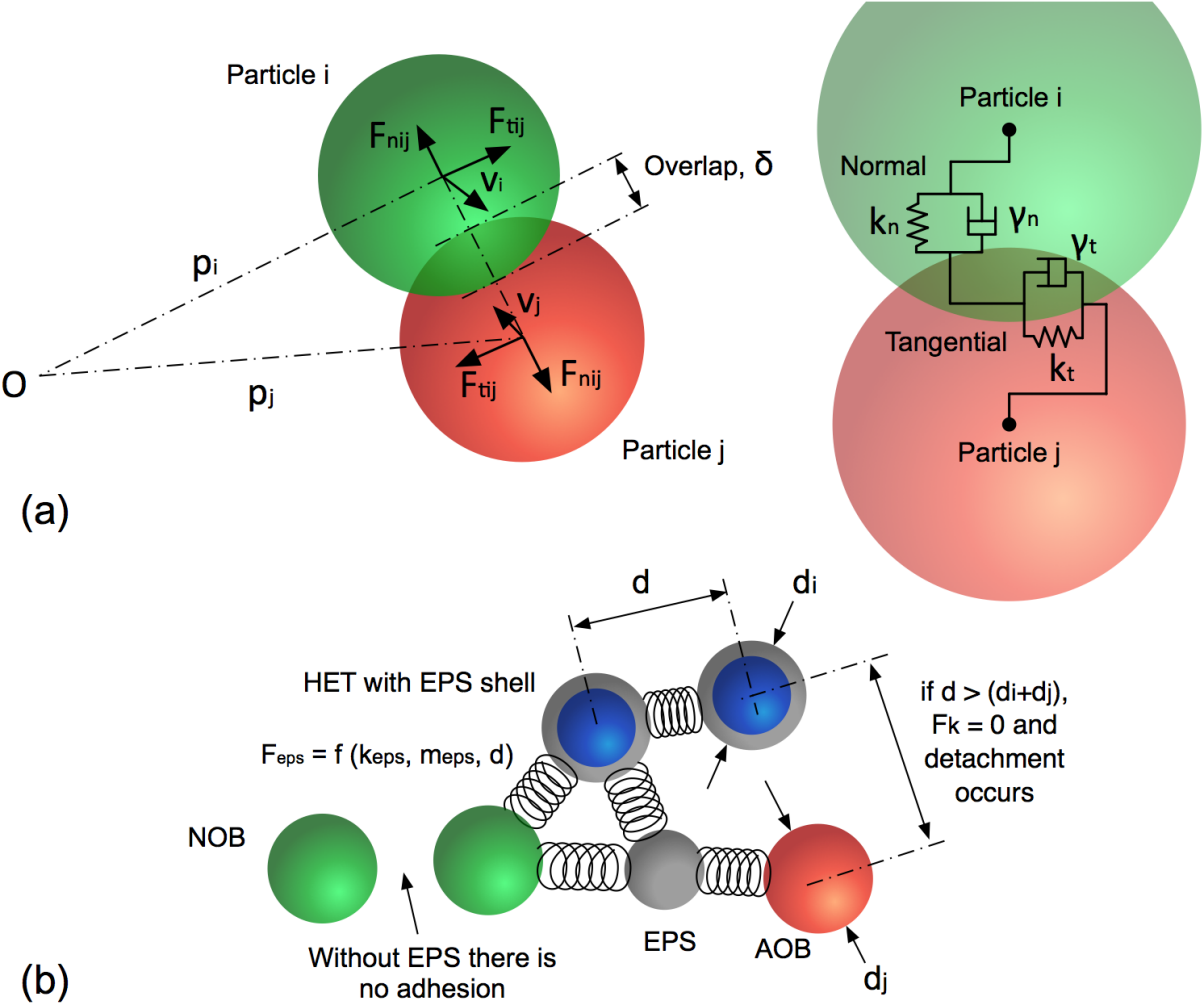
Mechanical interactions among the agents. (a) a schematic showing two particles *i* and *j* which are in contact, with their interaction governed by normal and tangential springs and dashpots. Particle location (*p*), velocity (*v*), overlap distance (*δ*), normal and tangential forces (*F*) are illustrated; (b) a schematic which shows agent-agent interactions through EPS mediated adhesive forces with EPS shown in grey.

The elastic, viscous and frictional contact forces between moving agents is modelled using a springs and dashpot model. The springs and dashpots are anchored at the centre of each particle (Fig 2A). The springs represents elastic interactions and the dashpot represents energy dissipation due to viscous forces between the two interacting agents. In the present work, a spring-dashpot model which is one of the default models in LAMMPS is employed for the normal (${\vec{F}}_{nij}$) and tangential (${\vec{F}}_{tij}$) forces acting on agent *i* due to interaction with agent *j* as given below.

$$\begin{array}{l} {\vec{F}}_{nij}=f(\delta_{ij})(k_{n}\delta_{ij}^{}{\vec{n}}_{ij}-\gamma_{n}m_{eff}^{}{\vec{v}}_{nij})\\ {\vec{F}}_{tij}=f(\delta_{ij})(-k_{t}{\vec{\delta }}_{tij}-\gamma_{t}m_{eff}^{}{\vec{v}}_{tij})\\ {\vec{F}}_{c,i}^{}=\sum_{j=1}^{N}({\vec{F}}_{nij}^{}+{\vec{F}}_{tij}) \end{array}$$ (4)

where *k*_n,t,_ *γ*_n;t,_ and ${\vec{v}}_{n,t}$ are the spring stiffness, viscous damping constant, and relative velocity between two agents, respectively; *m*_eff_ = *m*_i_*m*_j_ / (*m*_i_ + *m*_j_) is the effective mass of two agents with masses *m*_i_ and *m*_j_. The subscripts *n* and *t* represent the normal and tangential directions between the two agents. The corresponding contact force on particle *j* is simply given by Newton’s third law, i.e ${\vec{F}}_{ji}=-{\vec{F}}_{ij}$. The factor *f* (*δ*_ij_) = 1 is for a Hookean model (the linear spring-dashpot model), and *f* (*δ*_ij_) = $\sqrt{\delta_{ij}d}$ is for Hertzian contacts between two spheres. Here *δ*_*ij*_ is the overlap distance between two spherical agents *i* and *j* having diameters of *d*_*i*_ and *d*_*j*_, and *d* = 0.5*d*_*i*_*d*_*j*_/(*d*_*i*_ + *d*_*j*_). *N* is the total number of agents that interact with the *i*^*th*^ agent. The Hookean model is used for the present study. Detailed derivations of these well-established contact models of molecular dynamics can be seen in [Brilliantov et al. [36],Silbert et al. [37]], Plimpton [38]. The particle-wall contact forces are calculated in the same way as particle-particle interactions with the wall considered to be a particle with infinite radius. These contact models have previously been successfully deployed to simulate granular materials, where the microscopic physics plays a great role in determining the macroscopic response of the material subjected to an external force. The contact models are leveraged to capture the microscopic interactions between two microbes. Detailed temporal and spatial resolution of the microscopic interactions between each microbial pair are required for a fundamental understanding of the emerging biofilm morphology. The direct measurement of these physical model parameters can be challenging. However, these parameters could be calibrated based on well-designed experiments.

#### Adhesive force

The EPS adhesive force binding two agents is modelled as a spring, with the spring coefficient being proportional to the combined EPS mass of the two agents, i.e. the sum of the masses of the EPS shell(s) and/or EPS particles. If the total EPS mass of the two agents is $m_{ij}^{eps}$ and the spring coefficient per unit EPS mass is *k*^*eps*^, then the effective spring coefficient is calculated as $k^{eps}m_{ij}^{eps}$. The EPS force between two agents is calculated as the product between the effective spring coefficient and the separation distance between the two agents (Fig 2B). A similar approach was first suggested by Head [23] to produce a mechanically stable biofilm. The EPS-mediated binding forces are then calculated as:

$$\begin{array}{l} {\vec{F}}_{eps,ij}=k^{eps}m_{ij}^{eps}(d_{ij}-d_{0ij}).\frac{{\vec{d}}_{ij}}{d_{ij}}\\ {\vec{F}}_{a,i}=\sum_{j=1}^{N}{\vec{F}}_{eps,ij} \end{array}$$ (5)

Here *d*_*0ij*_ is the sum of the radii of two interacting agents and *d*_ij_ is the distance between centres of two agents. When the distance between two particles reaches 2 *d*_*0ij*_, the adhesive link breaks and the binding force becomes zero.

#### Drag force

The interaction of fluid and particulate agents is simplified by modelling one way coupling, i.e., only the effect of the fluid on the particle is considered, the flow field is not affected by the particles. In this work, the fluid drag force is based on Stokes flow past a sphere, and it is given by:

$${\vec{F}}_{f,i}=6\pi \mu \;r_{i}^{}\;{\vec{v}}_{r}$$ (6)

where $\mu ,r_{i},{\vec{v}}_{r}$ are the dynamic viscosity of fluid, radius of particle, and local fluid velocity relative to the particle, respectively. This force is zero only when there is no fluid flow field and the particle does not move relative to the surrounding fluid.

#### Model implementation in LAMMPS

The present IbM is implemented in the granular package in LAMMPS which supports interactions between finite size granular particles interacting with each other and with boundaries. Hertzian and Hookean contact models are embedded functions in this package. The IbM related features such as EPS-mediated binding forces, growth, division, nutrient transport etc. are new features that have been implemented in this package for this study. See Supporting information (S1 File) for details of how the IbM presented here interfaces with LAMMPS.

## Results and discussion

**Fig 3:**
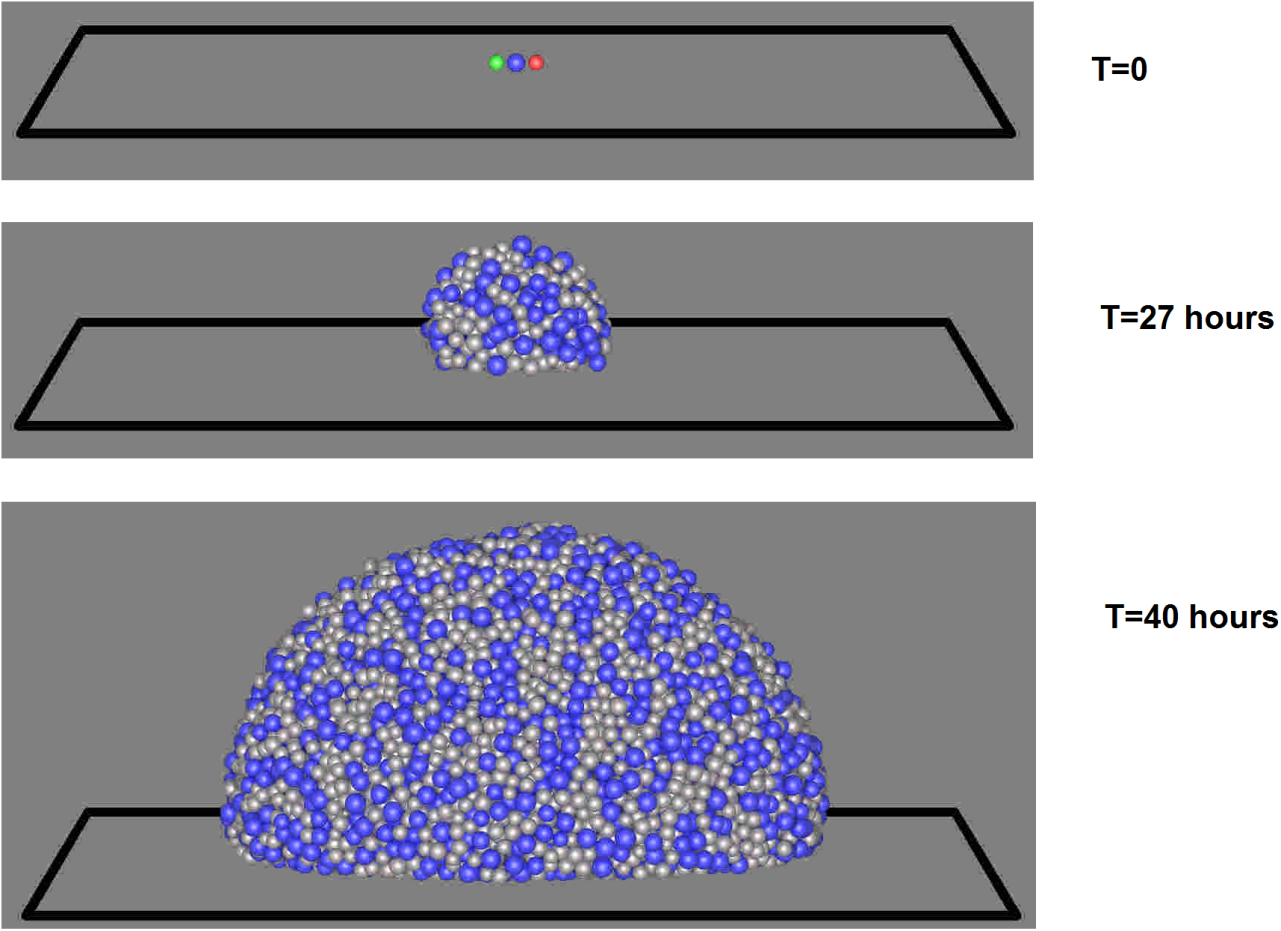
HET, AOB, and NOB interaction. It is seen that HET dominates in the biofilm when competing with AOB and NOB with typical parameters and the chosen nutrient conditions (red = AOB, green = NOB, blue = HET, grey = EPS).

For the simulations that follow, the computational domain (representative volume shown in Fig 1), [0,*L*_*x*_]×[0,*L*_*y*_]×[0,*L*_*z*_], has dimensions *L*_*x*_ = 100*μm*, *L*_*y*_ = 40*μm*, *L*_*z*_ = 100*μm*. The model is able to simulate both biofilms and flocs, but we have chosen to address biofilms in this study. As such, periodic boundary conditions are used in both *x* and *y* directions. The no-flux and Dirichlet boundary conditions for nutrients are used at the bottom substratum (*z* = 0) and the top boundary wall (*z* = *L*_z_), respectively. The number of grid points are *N*_*x*_ = 30, *N*_*y*_ = 12, *N*_*z*_ = 30. The diameter of the each bacterium in the inoculum is 1μm. To demonstrate how HET, AOB, and NOB grow and interact in the same environment, one bacterium from each functional group is initially inoculated at the centre of the substratum. When using typical parameters for each functional group and nutrient conditions [32,33], it is found that the growth of AOB and NOB is negligible comparted to HET and hence HET dominates in the biofilm (Fig 3). It is worth noting that HET could be outcompeted by AOB and NOB if the nutrients and initial inoculation were more favourable for AOB and NOB. As mentioned in the Method section, the mechanical equilibrium configuration of the biofilm is obtained when the growth-induced internal pressure due to overlap between agents becomes negligible as shown in Fig B in S1 File. It is seen that the growth-induced pressure releases over time and the biofilm reaches its equilibrium configuration within two seconds for this problem.

The model output has been assessed using a variety of case studies. For these simulations, different functional groups (AOB, NOB, and HET) are omitted for the sake of simplicity, and hence only a generic bacterial type (which is similar to HET), EPS, inert, and organic substrate are considered. The growth of the bacteria is limited by only the carbonic substrate (*S*_s_). Growth and morphology of this single species biofilm in quiescent and flow environments are studied. The decay of dead and EPS agents are assumed to be negligible compared to growth rates. This is to reduce the number of parameters involved in the simulations. The following first section addresses the influence of nutrient gradients on biofilm structure formation in a quiescent medium, the second section discusses the influences of a shear flow on a growing biofilm, and the third section offers insight into how a mature biofilm deforms and detaches due to fluid shear. The results are explained in terms of non-dimensional parameters which govern the nutrient transport (see S1 File). The physical parameters used for the following studies are given in Table 1. The initial inoculation has 40 numbers of bacterial cells which are evenly distributed on the substratum. Each simulation is run for five replicates and the average results are calculated.

**Table 1:** Input parameters for biofilm simulations.

Parameters	Symbol	value	Unit	Reference
*Computational domain*				
Dimensions	*L*_*x*_×*L*_*y*_×*L*_*z*_	100×40×100	μ m^3^	Chosen
Cartesian grid cells	*N*_*x*_×*N*_*y*_×*N*_*z*_	30×12×30	-	Chosen
*Kinetic and stoichiometric*				
Maximum specific growth rate	*μ*_*m*_	1	h^-1^	[40]
Decay coefficient	*b*	1.33×10^−2^	h^-1^	[56]
Substrate affinity	*K*_*S*_	3.50×10^−5^	kg m^-3^	[40]
Yield coefficient	*Y*	0.61	gcod/gcod	[57]
EPS formation coefficient	*Y*_*EPS*_	0.18	gcod/gcod	[57]
*Mass transfer*				
Diffusion coefficient for substrate	*D*_*S*_	1.6×10^−9^	m^2^ s^-1^	[40]
Bulk substrate concentration	*S*_*b*_	1×10^−4^	kg m^-3^	Chosen
*Mechanics*				
Spring coefficient for collision	*k*_n_	1×10^−4^	N m^-1^	[24]
Viscous coefficient for collision	*γ*_*n*_	1×10^−5^	s^-1^	Chosen
EPS stiffness per unit EPS mass	*k*^*e*^	5×10^9^	s^-2^	[23]
Dynamic viscosity	*μ*	1×10^−3^	Pa s	For water

### Effect of growth parameters on biofilm structure

For a simple system with only one bacterial species and one nutrient, without fluid flows, non-dimensionalization of the diffusion-reaction and biomass growth equations (see S1 File) yields three non-dimensional parameters which determine the nutrient gradients across the biofilm:

$$\delta =\sqrt{\frac{S_{b}D_{S}Y_{}}{\mu_{m}\rho_{X}L_{z}{}^{2}}};\;\kappa =\frac{K_{S}}{S_{b}};\;\beta =\frac{b_{}}{\mu_{m}}$$ (7)

where *S*_*b*_ and *D*_*S*_ are the bulk nutrient concentration and its diffusivity, respectively; *μ*_*m*_ and *b* are the maximum specific growth rate and decay rate of bacteria, respectively; *K*_*S*_ is the substrate affinity to bacteria, *Y* is yield coefficient for bacteria growth, and *ρ*_*X*_ is the biomass density.

**Fig 4:**
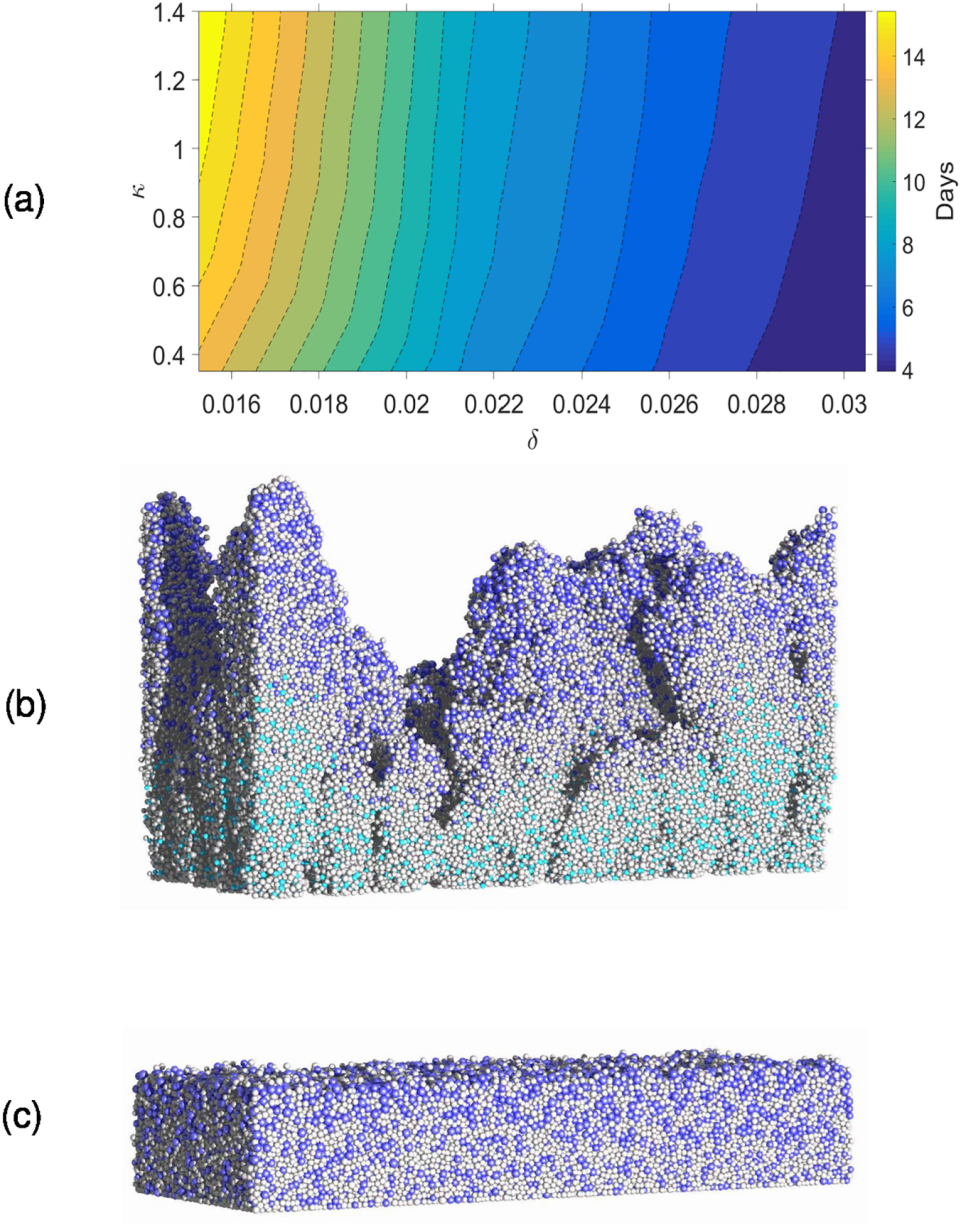
Biofilm growth under different nutrient conditions. (a) average number of days to reach pre-defined volume; (b) biofilm with the lowest values of *δ* and *κ*; (c) biofilm with the highest values of *δ* and *κ* (colour code for cells: blue = bacteria, cyan = inert, grey = EPS).

**Fig 5:**
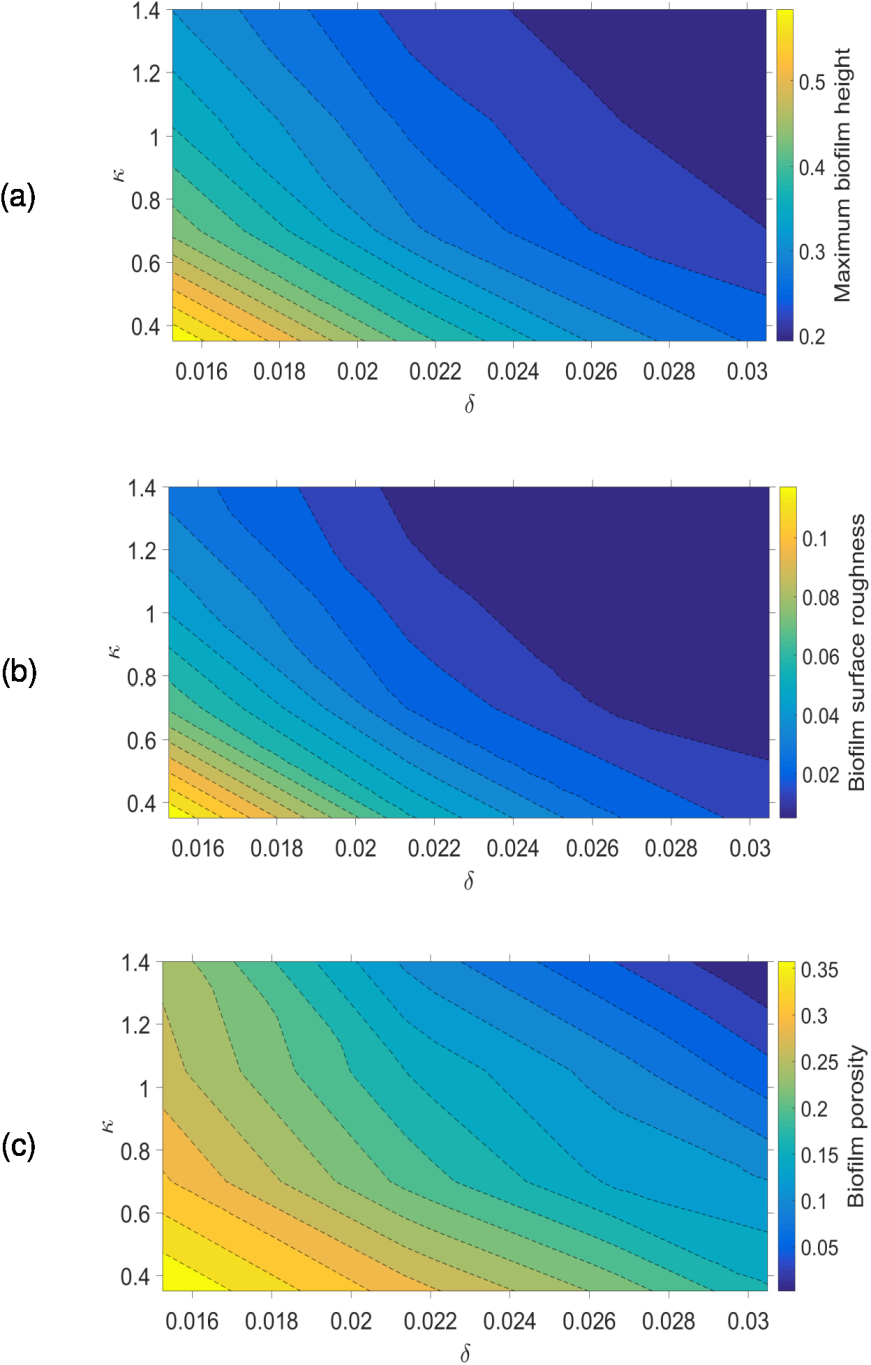
Variation of biofilm emergent properties with *δ* and *κ*. (a) maximum height; (b) RMS surface roughness; (c) porosity. The biofilm structure is equally dependent on *δ* and *κ*.

As already discussed in the literature [11,39], the parameter *δ* represents the ratio between the maximal nutrient transport to the biofilm and the maximal nutrient consumption by the bacteria. Parameter *κ* is the non-dimensional Monod coefficient and *β* is the relative decay rate of biomass. The emergent properties of the biofilm are studied for a range of *δ* and *κ* values while *β* is kept constant. The values of *δ* and *κ* are varied independently by changing the nutrient concentration and Monod saturation coefficient. The smallest values of *δ* and *κ* are based on the parameters in Table 1 and the largest values are four times the smallest values (i.e., *δ* = 1.52×10^−2^ to 3.05×10^−2^; *κ* = 0.35 to 1.40; *β* = 1.33×10^−2^). The biofilm morphology is characterised by average height, root-mean-square (RMS) surface roughness, and biofilm overall porosity, calculated as follows:

$$\begin{array}{l} \mathrm{average}\;\mathrm{height}=\bar{h}=\frac{1}{L_{x}L{}_{y}}\iint h(x,y)dxdy,\\ \mathrm{roughness}={\left(\frac{1}{L_{x}L{}_{y}}\iint {\left(h(x,y)-\bar{h}\right)}^{2}dxdy\right)}^{1/2},\\ \mathrm{porosity}=1-\frac{\sum_{i=1}^{N_{X}}\sum_{j=1}^{N_{Y}}\sum_{k=1}^{N_{Z}}F(i,j,k)}{N_{X}N_{Y}h_{\max}}, \end{array}$$ (8)

where *h*(*x*, *y*) is the biofilm height (measured in *z* direction) at location (*x*, *y*) on the substratum. *F*(*i*,*j*,*k*) is equal to 1 if the grid voxel (*i*, *j*, *k*) is filled by biomass and zero otherwise. These biofilm characteristics are calculated when the biofilm reaches a pre-defined volume (15% of the simulation box is chosen in this study). The time taken to reach this volume varied between 4 and 16 days depending on *δ* and *κ* (Fig 4). It is clear that the growth duration is negatively correlated (that is, growth rate is positively correlated) with *δ* and *κ* and the biofilm growth rate is more sensitive to *δ* than *κ* for the selected parameter range. For the lowest values of *δ* and *κ*, the biofilm surface is very rough and for the highest values of *δ* and *κ* the biofilm is compact and its surface is smooth. For rougher biofilms, water channels between finger-like biofilm structures are evident. The biofilm height, surface roughness and porosity increase when *δ* and *κ* decrease (Fig 5), and this is consistent with other simulations in the literature [11,23,39]. The results indicate that the biofilm structure is equally dependent on *δ* and *κ*.

As already mentioned, *δ* represents the ratio of the maximum nutrient uptake rate to the maximum nutrient consumption rate. Similarly, *κ* is an inverse measure of bacterium affinity to organic substrate. Even though the biological meaning of *κ* is not intuitively obvious, *κ*^−1^ can be considered as a representation of how easily nutrients are transported across a bacterial membrane. It is obvious from the diffusion-reaction equation that the nutrient gradients would be negatively correlated with *δ* and *κ* (see S1 File), which means that the nutrient gradients decrease as either parameter increases. This also means that an increase in either parameter results in nutrient rich conditions throughout the biofilm and therefore most of the cells in the biofilm would grow significantly when *δ* and *κ* increase. However, when *δ* and *κ* decrease, the nutrient gradients increase which depletes nutrients at the bottom of the biofilm leaving only the bacteria near to the top surface active, resulting in slower biofilm growth. The top layer of the biofilm where the bacteria are growing rapidly is commonly referred to as the ‘active layer’. As discussed in Nadell et al. [39], *δ* can be considered to be the non-dimensional active layer thickness and hence as *δ* decreases the number of active bacteria decreases. Head [23] calculated the active layer thickness by determining which cells are active based on their growth rates, and has shown that active layer thickness is negatively correlated with biofilm roughness. Fig C in S1 File shows the growth rates of individual cells and variation of active layer thickness over time at very low nutrient conditions (*δ* = 1.52×10^−2^ and *κ* = 0.35). The active layer can be distinguished from the rest of the biofilm, and it can be seen that the average active layer thickness is approximately constant over the time. The lower number of active cells together with the stochastic nature of cell division produces some very rough biofilm shapes for smaller *δ* and *κ* values.

**Fig 6:**
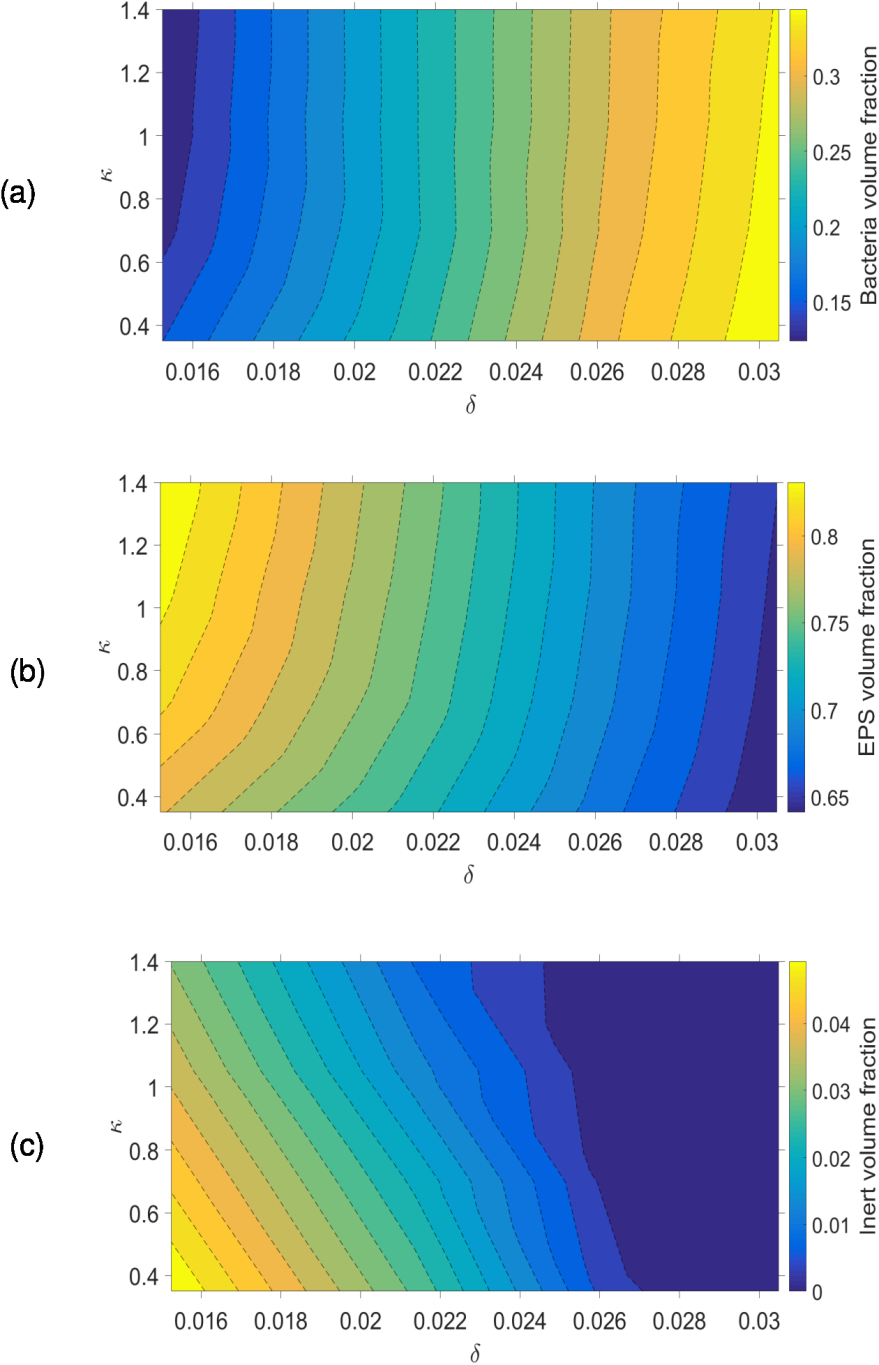
Variation of volume fractions with *δ* and *κ*. (a) bacteria; (b) EPS; (c) inert. It is seen that the biofilm composition is most sensitive to changes in *δ*.

The biofilm composition varies with *δ* and *κ* (Fig 6), and is more sensitive to variations in *δ* than *κ*. The bacteria volume fraction increases with *δ*; when *δ* increases more nutrient can penetrate into the biofilm increasing bacterial activity. The dead cell volume fraction declines to zero when *δ* increases because bacteria then grow in nutrient-rich environments (Fig 6C).

**Fig 7:**
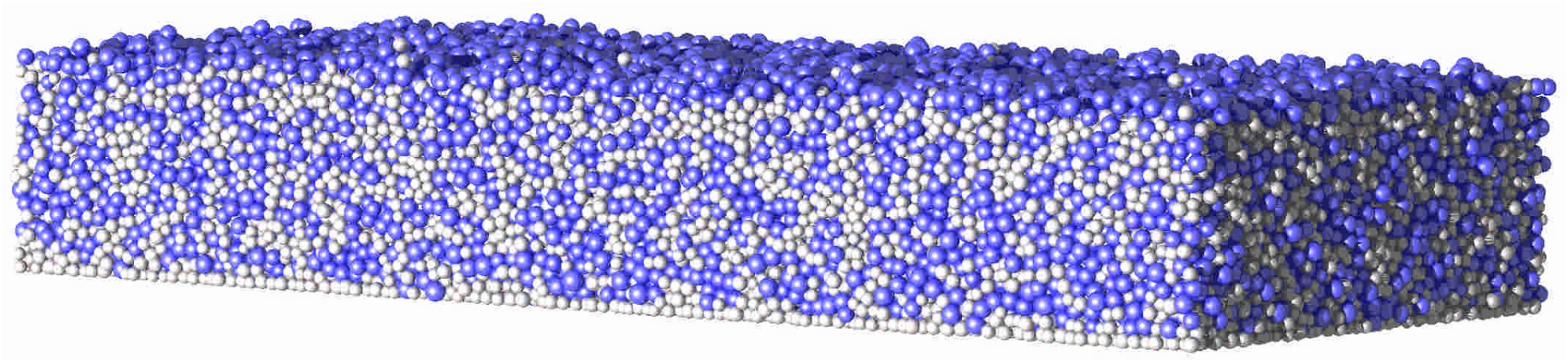
Biofilm growth when nutrients are supplied from the bottom substratum instead of the top wall. The same *δ*, *κ*, and *β* values as in Fig 4(B) are used for this simulation. The biofilm surface is flat in this case when compared with Fig 4(B) where the nutrient is supplied from the top.

It is worth noting that the biofilm structure is also influenced by the direction in which the nutrients are supplied to the biofilm, i.e. from the top or bottom. To demonstrate this effect, the biofilm is grown at the smallest *δ* and *κ* values (i.e., *δ* = 1.52×10^−2^; *κ* = 0.35; *β* = 1.33×10^−2^) with nutrient supplied from the bottom wall which is the substratum (similar to biofilm growth on an agar plate). This results in a smooth and compact biofilm (Fig 7), caused by the close proximity of the source of nutrients and biofilm surface, giving reduced nutrient depletion inside the biofilm in comparison to the biofilm in Fig 4(B). Moreover, if nutrients are provided from the bottom, there are no preferential nutrient gradients in the fluid medium which result in formation of biofilm ‘towers’. That would also be a reason for the flat and compact biofilm obtained in this case. This finding is consistent with the results in Schluter et al. [40].

### Biofilm growth in simple shear flow

In the previous section, it was seen that decreasing nutrient availability (decreased *δ* and *κ* values) results in increased biofilm porosity and a tendency towards columnar structures in the biofilm when grown in a still medium. However, if the biofilm grows in a moving fluid the structural properties of the biofilm will differ [41]. Experimental work has shown that high shear forces result in thinner, denser, stronger and filamentous biofilms while low shear forces result in biofilms with mushroom and circular shapes [42]. It is also reported in the literature that biofilms produce undulating shapes when grown in flow fields [23,42].

**Fig 8:**
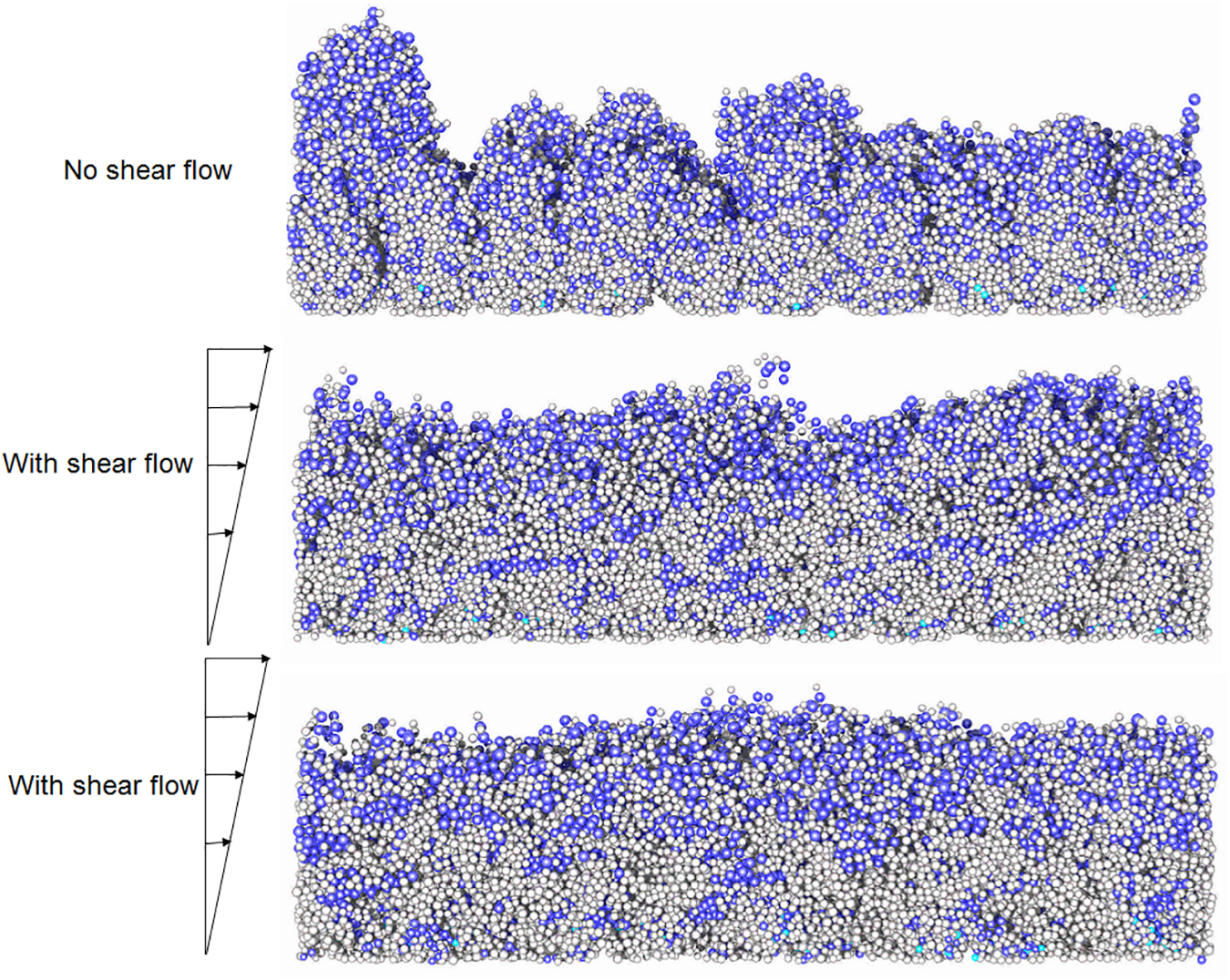
Biofilm growth under a shear flow. The biofilm shapes are shown at the non-dimensional time T^*^ = 140 (T* = *t*(*s*)×*μ*_*m*_(*s*^−1^)); (a) $\dot{\gamma }/\mu_{m}=0$, very rough shape; (b) $\dot{\gamma }/\mu_{m}=108$, wavy pattern; (c) $\dot{\gamma }/\mu_{m}=144$, flat shape. As shear rate increases the biofilm surface changes from very rough to wavy and then smooth.

**Fig 9:**
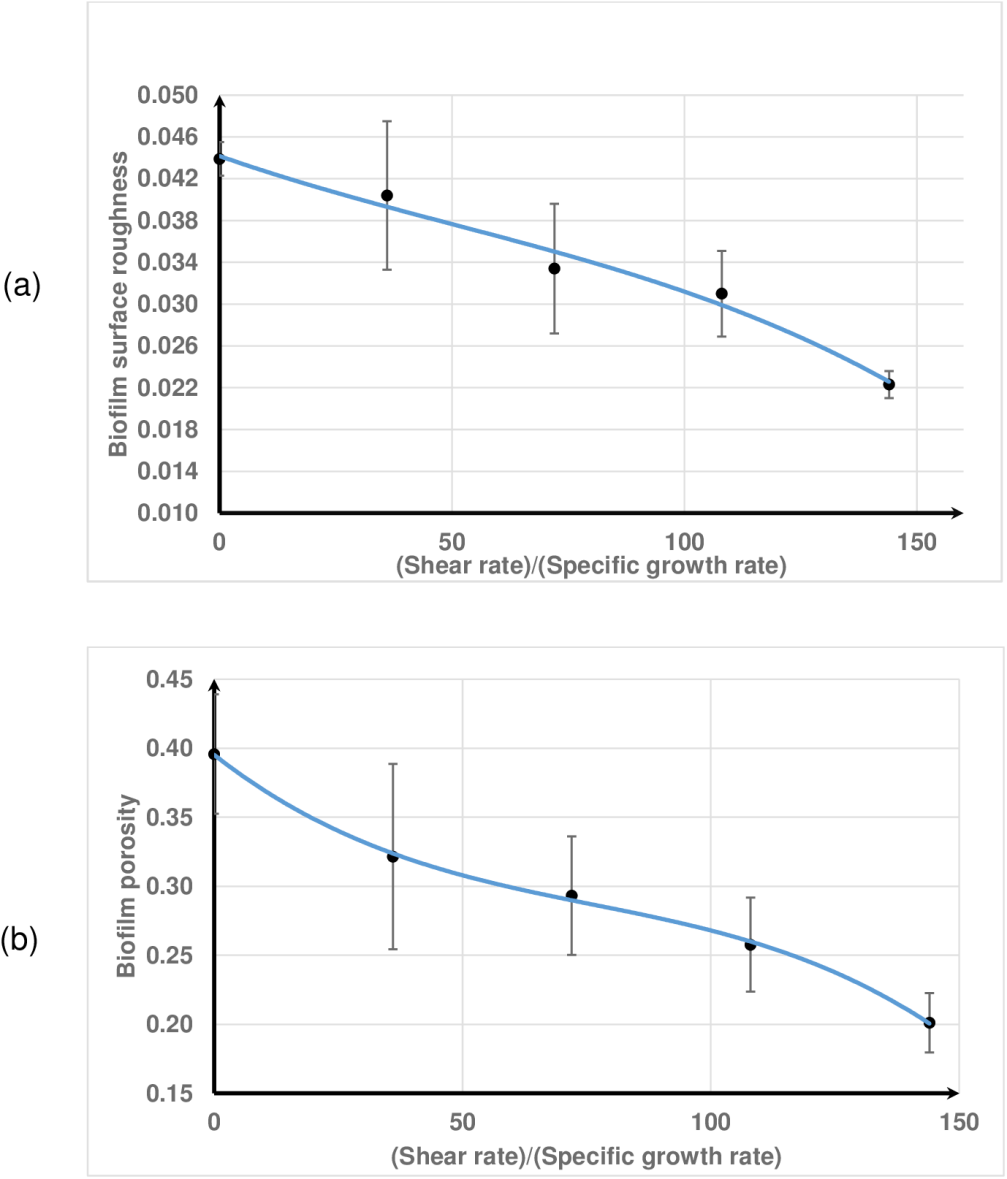
Biofilm emergent properties at time T^*^ = 140. (a) surface roughness; (b) porosity (error bars show ± 1 standard deviation calculated from five replicates).

Biofilm growth has been simulated subject to simple shear flow in the x direction at a shear rate $\dot{\gamma }$. The flow velocity is linearly varied from zero at the substratum (z = 0) to the maximum velocity at the top wall (z = *L*_z_). The shear rate is varied between 0 and 0.04s^-1^ and the resulting biofilm structure is observed (Fig 8). As the shear rate increases the shape of the biofilm changes from very rough to wavy, and then to a smooth surface at high shear rates (S1 and S2 Videos). The simulation predicts that biofilm roughness and porosity decrease with increasing shear rate (Fig 9) which is consistent with literature. When the shear flow is strong enough the biofilm protrusions detach and then, because of the periodic boundary conditions, re-attach on to the growing biofilm and hence the biofilm becomes smoother at higher shear rates with these boundary conditions.

### Biofilm deformation and detachment in simple shear flow

The presence of biofilms in different systems such dental plaque, contact lenses, catheters, medical implants, pipes etc. cause economic losses and threats to public health [43]. Removal of these biofilms can be achieved by the application of hydrodynamic shear forces [44,45]. The mechanical response of biofilms to shear flow principally derive from the cell-cell interactions and EPS-cell interactions implemented in this model. Therefore, it is interesting to study the emerging mechanical responses of the bulk biofilms, which will advance the understanding of viscoelastic properties and detachment of biofilms. To simulate hydrodynamic biofilm removal, a shear flow is applied to a pre-grown biofilm. Shear-induced detachment of these biofilms can be divided into two main types: continuous detachment of single cells or small cell clusters which is called *erosion*, and detachment of large clusters of biomass which is called *sloughing* [46]. In existing Ib models, bacterial detachment is typically implemented by specifying assumed detachment probability functions or detachment rate functions rather than use of mechanistic approaches [19,30,47]. The IbM developed in this study enables us to predict biofilm deformation, detachment and streamer formation based on mechanical interactions between EPS and cells. As a case study, four bacterial cells are inoculated on the substratum and the biofilm is initially grown to a certain height without flow (at *δ* = 1.52×10^−2^; *κ* = 0.35; *β* = 1.33×10^−2^). Then, shear flow is applied to the biofilm to study detachment from the mature biofilm (this is different from the previous section where both shear and growth occur simultaneously). The effect of shear rate and initial biofilm height on biofilm detachment is studied. The computational domain is doubled in the *x* direction (i.e., *L*_*x*_ = 200*μm*) so that detached clusters can be tracked as they move away from the biofilm.

#### Effect of shear rate

**Fig 10:**
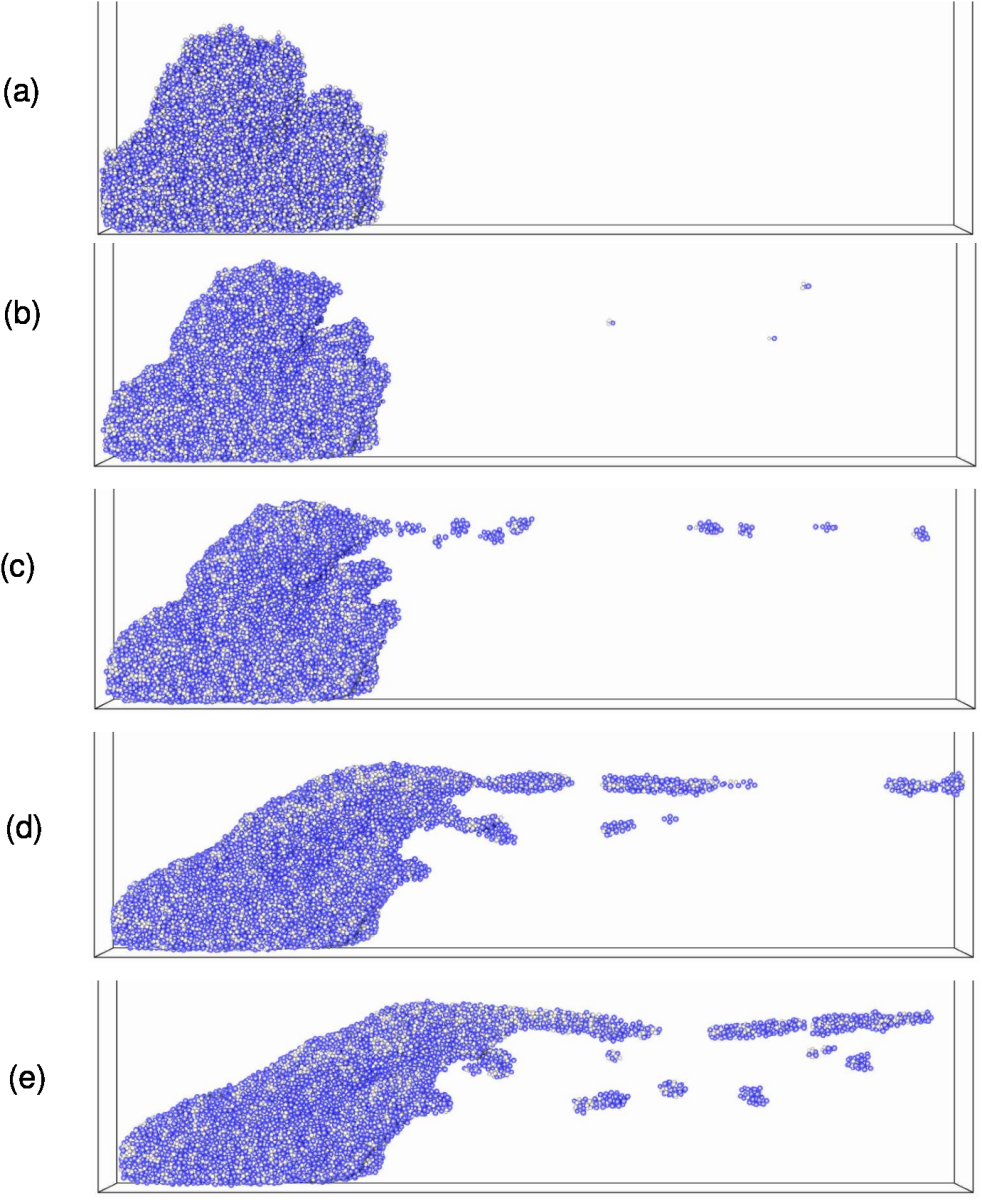
Biofilm deformation and detachment at $\dot{\gamma }=0.20s^{-1}$. (a) time, T^**^ = 0; (b) T^**^ = 8000; (c) T^**^ = 20000; (d) T^**^ = 70000; (e) T^**^ = 130000. Streamer formation and detachment is evident at the later stages of the deformation.

A biofilm is grown for 4.6 days until it reaches a height of 48μm and then simple shear flow is applied. Detached biomass exits the simulation box at the right-hand boundary. Six different shear rates, 0.04, 0.08, 0.12, 0.16, 0.20, and 0.24 s^-1^, are applied to the pre-grown biofilm. Fig 10 and S3 Video show the initial biofilm grown under static flow conditions, deformation and subsequent detachments at $\dot{\gamma }=0.20s^{-1}$ (here the non-dimensional time, T^**^, is calculated as real time (*s*) × shear rate (*s*^*-1*^), $T^{**}=t(s)\times \dot{\gamma }(s^{-1})$). The biofilm initially deforms in the direction of flow and bacteria detach from the downstream side of the biofilm. These bacterial detachments are discrete events which occur throughout the simulation, and could be described as erosion because the cluster sizes are at least an order of magnitude smaller than the biofilm (for sloughing events the typical cluster sizes could be in the same order of magnitude as the biofilm). Three phases can be identified in the detachment process. In the first phase, the biofilm rapidly deforms in the flow direction and very small clusters erode from the deforming biofilm (Fig 10B). In the second phase, deformation continues but the detached clusters increase in size (Fig 10C). These clusters detach from the moving top of the biofilm due to cohesive failures. In the third phase, the top of the biofilm is highly elongated in the flow direction to become a filamentous streamer, which subsequently detaches (Fig 10D and 10E), creating larger detached clusters in the flow field. Some of the detached clusters break up again in the flow field and re-agglomerate with one another (see S3 Video). These breakup and agglomeration events are commonly observed in activated sludge process [48] due to floc collisions and shearing.

**Fig 11:**
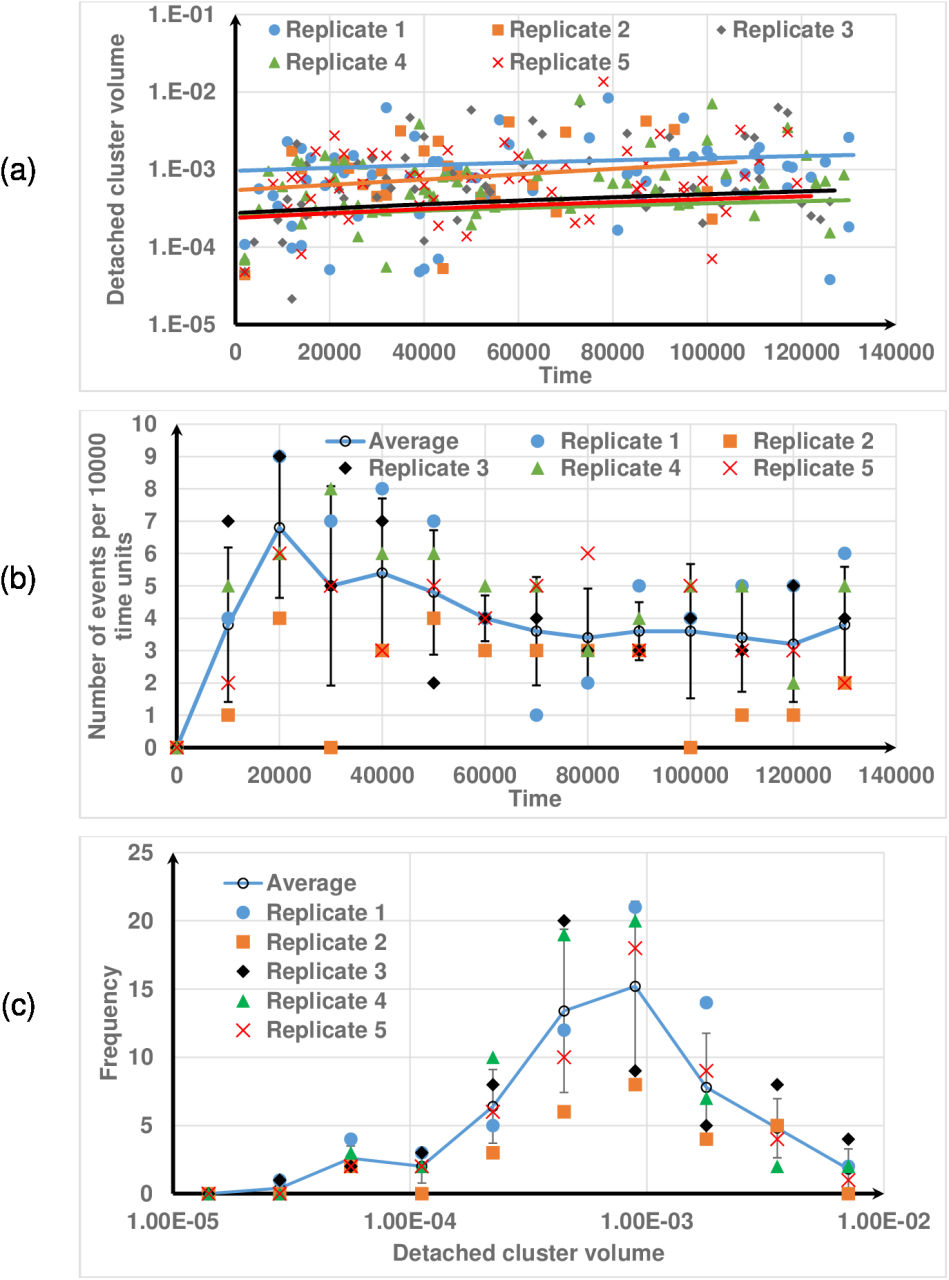
Biofilm detachment at $\dot{\gamma }=0.2s^{-1}$. (a) detachment cluster volume with time; (b) number of events with time; (c) distribution of detached cluster volume. The error bars show ±1 standard deviation calculated from five replicates. Results from all replicates are shown to demonstrate that they all follow the same trend.

Fig 11(A) shows the transient distributions of the volumes of detached clusters for different replicates at $\dot{\gamma }=0.20s^{-1}$, with the biofilm volume reducing over time due to biomass detachment. The volume of each detached cluster has been normalized by the remaining biofilm volume. Different replicates give slightly different initial biofilm shapes and spatial distributions of EPS due to the stochastic nature of the biofilm growth. This results in different size distributions of detached clusters even though the detachment algorithm is deterministic. The linear trend lines show that the average size of the clusters increases slightly over the time that the shear flow is applied, but there is much greater variation at any given time point with the size of the detached clusters varying by several orders of magnitude (10^−5^ to 10^−2^) (Figs 10 and 11). The number of detachment events per unit time increases to a maximum value and then gradually decreases with time (Fig 11B). It should be noted that all of the replicates show this trend. Initially the biofilm deforms with little detachment, and then as the inter-particle distances reach their limiting value the number of detachment events significantly increases (Fig 10 and S3 Video). With prolonged shear flow, local shear stress exceed the shear threshold in multiple sites, resulting in increased detachment events and larger detached clusters. Depending on the shear rate and viscoelastic properties of the biofilm, biofilm streamers may form which lead to a reduction in the number detachment events in the later stages of the simulation. Like many other soft biological materials, the deformation of biofilms is time-dependent due to its viscoelastic nature [49,50]. All these observations are fairly consistent with experimental work. For example,Walter et al. [51] have demonstrated that when a shear flow is applied to a mature biofilm, the number of erosion events suddenly increases and then gradually decreases over the time. Fig 11(C) shows the frequency distribution of the volume of the detached clusters from time T^**^ = 0 to 1.3×10^5^. It is evident that the detached cluster sizes have a lognormal distribution. The maximum and mean volumes of the clusters are respectively 0.8% and 0.07% of the initial biofilm volume. The total biomass detached is about 6.5% of the initial biomass. These figures suggest that the detachment events occurring here are erosion rather than sloughing because detached masses are relatively small. The results in Fig 11 also indicate that the stochastic variation of the initial biofilm shape and spatial distribution of EPS significantly affects the number of events and the cluster size distributions since the standard deviations are large.

**Fig 12:**
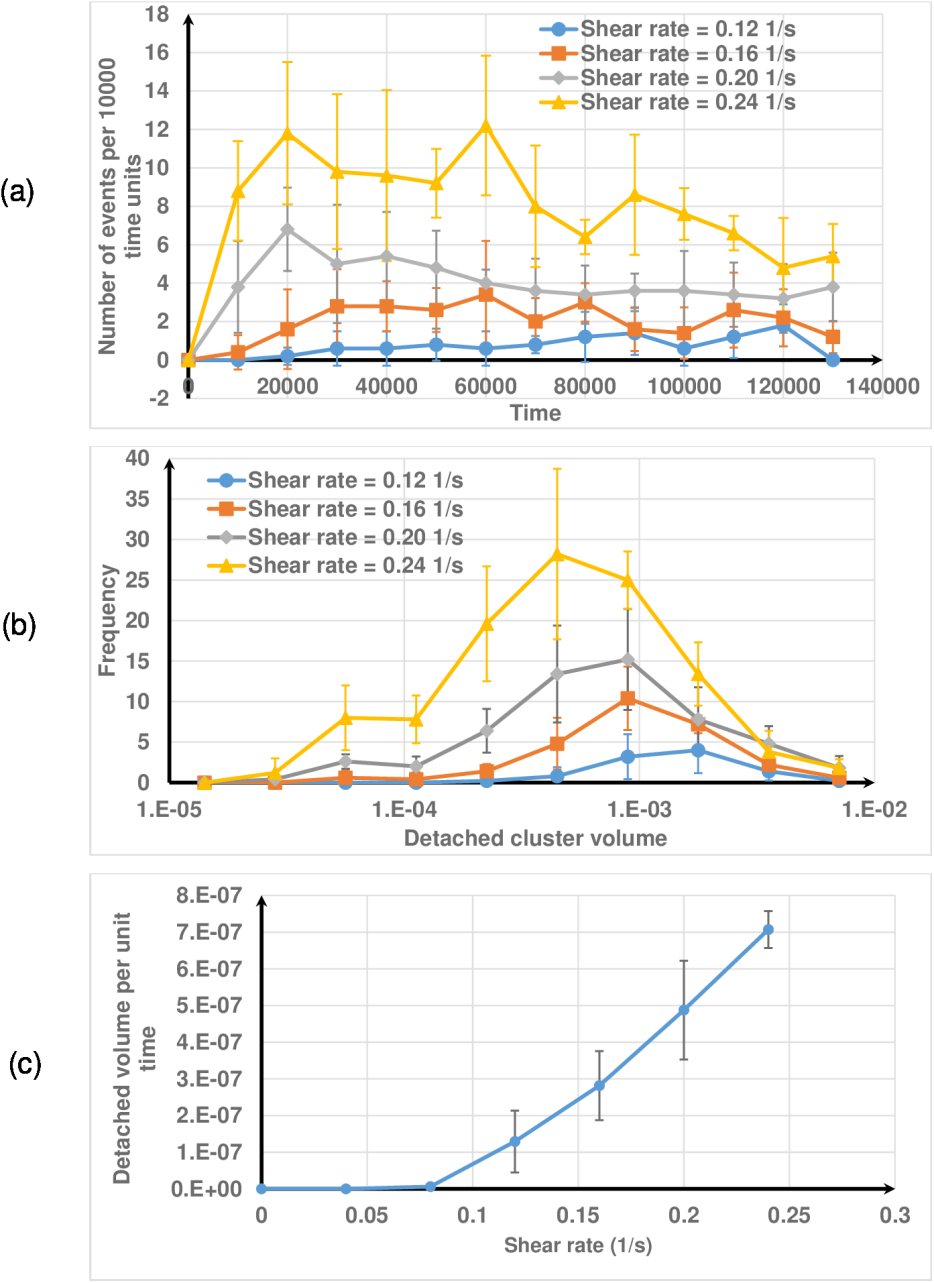
Effect of shear rate on biofilm detachment. (a) number of events; (b) distribution of the volumes of detached clusters; (c) detachment rate, calculated as (total detached volume)/(total time). The mean volume of detached clusters decreases as shear rate increases.

The number of detachment events per unit time increases initially and is then fairly constant for smaller shear rates (0.12, 0.16 s^-1^) (Fig 12A). As expected, when the shear rate increases the number of detachment events also increases. The results demonstrate that if the shear rate increases many detachment events occur at the beginning of the process and then gradually decrease. Extended streamers develop at higher shear rates (Fig D in S1 File) and this reduces the frequency of detachment events in the later stages since they take a longer time to detach. However, at lower shear rates, elongated streamers do not develop, and instead, clusters detach from the top of the biofilm. The frequency distribution of detached cluster volume moves leftward as shear rate increases, indicating that the average cluster volume decreases as shear rate increases (Fig 12B). Even though larger clusters are produced at higher shear rates due to streamer detachment, that frequency is smaller. Walter et al. [51] also reported that the mean cluster size for erosion would decrease as shear rate increases. As expected, there is a monotonically increasing relationship between the average detachment rate and shear rate (Fig 12C). This is attributed to the fact that elastic deformation and detachment (localised cohesive failure) will dominate at very high shear rates with little energy dissipation due to viscous effects (or damping effects), as the energy dissipation by dashpot is time-dependent and lags behind elastic deformation. Such stress rate dependent failure behaviour was also observed in other non-living thin films [52].

#### Effect of biofilm height

**Fig 13:**
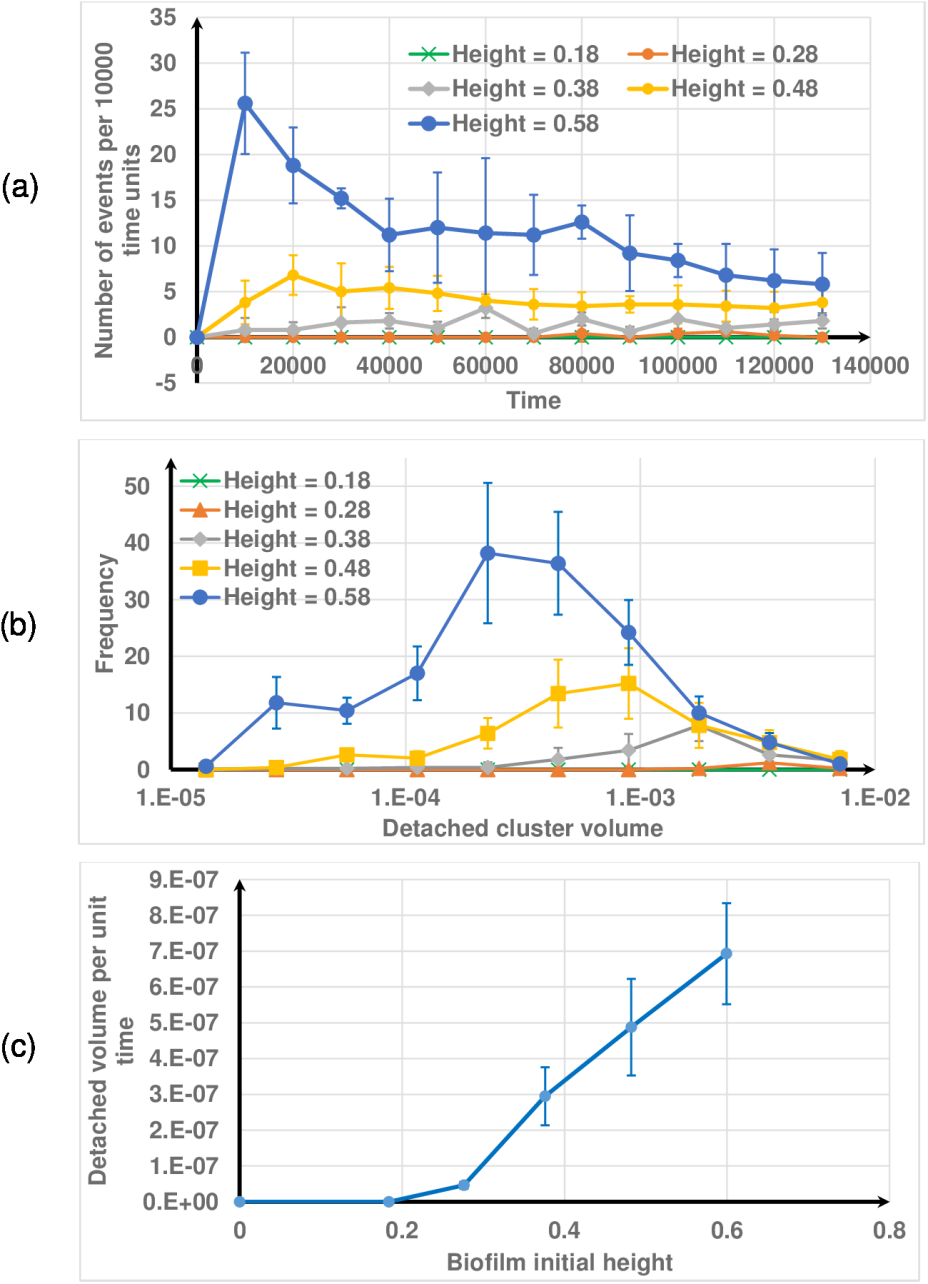
Effect of biofilm height on biofilm detachment. (a) number of events; (b) distribution of the volumes of detached clusters; (c) detachment rate. The mean volume of detached clusters decreases as height increases. The initial biofilm height is shown in non-dimensional form.

Next, the effect of initial biofilm height on detachment behaviour is investigated under constant shear rate ($\dot{\gamma }=0.2s^{-1}$). Different initial biofilm heights (18, 28, 38, 48 and 58 μm) are obtained by growing the biofilm for different durations. The effect of initial biofilm height on detachment is similar to the effect of varying the shear flow (Fig 13). This is expected since the fluid velocity experienced by any particle is the product of the shear rate and the distance between the particle and the substratum. As such, similar effects on the drag force is obtained by varying the shear rate and keeping the initial height constant and *vice versa* (i.e., constant shear and varied height).

When comparing the empirical detachment models for IbM which have appeared in the literature [6], most of those models are in the form “*detachment rate* = $k_{1}\mu_{m}^{k_{2}}\rho_{X}^{k_{3}}L_{F}^{k_{4}}\tau^{k_{5}}$”, where *k*_1_-*k*_5_ are constants, *L*_F_ is the biofilm height and *τ* is the shear stress.

Fig 12(C) shows that the rate of detachment increases at increasing rate with shear rate. As such, Fig 12(C) indicates that detachment rate would be proportional to $\tau^{k_{5}}$ because the shear stress *τ* is proportional to the shear rate $\dot{\gamma }$ for Newtonian fluids. Fig 13(C) also shows that the detachment rate seems to be proportional to $L_{F}^{k_{4}}$ and it suggests that the mechanical based model we present here will be an appropriate tool for biofilm detachment simulations.

Analogous results (streamer formation and subsequent detachment of biomass in shear flow) are reported in the literature in studies using other mechanistic approaches such as immersed boundary [53], dissipative particle dynamics [54] and phase field [55] methods. These works employ a more accurate two way coupling between biofilm and fluid. However, emergence of spatial distribution of EPS due to mechanical interactions between EPS and cells and analysis of biomass detachment based on EPS-cell adhesion are lacking in these works. The incorporation of two-way coupling is a logical and feasible next step in the development of the present model.

### Conclusions and future work

The properties of microbial communities are a function of interacting biological, chemical and physical processes. Thus if we are to understand and predict these properties we must strive to include all significant processes in a model. The primary objective of the current version of the model was to implement an IbM of microbial communities in LAMMPS with physics based mechanical interactions. This is the first application of the open source simulator LAMMPS to model microbial communities. It has already demonstrated the advantage in terms of prediction of flow rate dependent biofilm deformation, detachment, and streamer formation, which cannot be achieved using existing IbM software. The results for biofilm structure formation show good qualitative agreement with previous simulations and experimental results. This is the first IbM to predict erosion and streamer formation as emergent biofilm properties based on agent-agent mechanical interactions and EPS-mediated adhesion. It has demonstrated that this modelling approach enables prediction of growth and deformation of bacterial biofilms at micro-scale.

The advantages of implementing the IbM in LAMMPS are that it is:

- open source,
- capable of modelling advanced mechanical interactions (enabling more complex mechanical interactions to be implemented in future models),
- modular, permitting the addition of further biological and physical processes,
- frequently employed on high performance computers (HPC) using massively parallel simulation.

The present model is the first step towards an advanced Ib model and the key future steps may include a more sophisticated IbM which will incorporate other features including physicochemical reactions, pH, temperature, pili-mediated binding forces, quorum sensing, and bacteria motility. Full hydrodynamic coupling will be implemented by linking the present LAMMPS model to OpenFOAM, an open-source fluid modelling platform [27]. The present version of the model is also run in serial. However, we will fully exploit its merit in terms of massively parallelization of the model. The MPI parallelism of the particle-based functions such as growth, division, mechanical interactions will be based on LAMMPS parallel mechanism. Once our LAMMPS Ib model is coupled with OpenFOAM, the nutrient and fluid solvers will be then handled on OpenFOAM. Computational efficiency of the model will be further discussed after we improve the efficiency of the model.

## Supporting information

S1 File Supporting information (SI). (DOCX)

S1 Video Growth of a biofilm without flow. If there is no flow field the shape is determined by the nutrient level.
(WMV)

S2 Video Growth of a biofilm at a shear rate of 0.01 s^-1^. If the biofilm grows in a flow field, the shape is determined by both nutrient level and flow fields.
(WMV)

S3 Video Biofilm growth, deformation, and detachment at shear rate of 0.2 s^-1^. The biofilm is initially grown for 4.6 days without flows and then shear flow is applied on the grown biofilm for another 4.6 days. It is seen that the detached clusters agglomerate with one another.
(WMV)
