# Supplementary material for: A mechanistic Individual-based Model of microbial communities
Source: PLoS One. 2017 Aug 3;12(8):e0181965. doi: 10.1371/journal.pone.0181965 (PMC5542553; doi:10.1371/journal.pone.0181965)
Supplement: S1 File — (DOCX) [file pone.0181965.s001.docx]

**Supporting information (SI)**

**A Mechanistic Individual-based Model of Microbial Communities**

Pahala Gedara Jayathilake^1*^, Prashant Gupta^2†^, Bowen Li^3^, Curtis Madsen^3‡^, Oluwole Oyebamiji^4^, Rebeca González-Cabaleiro^5^, Steve Rushton^2*^, Ben Bridgens^6^, David Swailes^1^, Ben Allen^6^, A. Stephen McGough^3^, Paolo Zuliani^3^, Irina Dana Ofiteru^5^, Darren Wilkinson^4^, Jinju Chen^1*^, Tom Curtis^6*^

^1^ School of Mechanical & Systems Engineering, Newcastle University, Newcastle upon Tyne, NE17RU, UK

^2^ School of Biology, Newcastle University, Newcastle upon Tyne, NE17RU, UK

^3^ School of Computing Science, Newcastle University, Newcastle upon Tyne, NE17RU, UK

^4^ School of Mathematics & Statistics, Newcastle University, Newcastle upon Tyne, NE17RU, UK

^5^ School of Chemical Engineering and Advanced Materials, Newcastle University, Newcastle upon Tyne, NE17RU, UK

^6^ School of Civil Engineering & Geosciences, Newcastle University, Newcastle upon Tyne, NE17RU, UK

* Corresponding authors: jayathilake.pahala-gedara@ncl.ac.uk; steven.rushton@ncl.ac.uk; [tom.curtis@ncl.ac.uk](mailto:tom.curtis@ncl.ac.uk); jinju.chen@ncl.ac.uk

† Now at Procter & Gamble Technical Centres Ltd, Newcastle Upon Tyne, UK

‡ Now at Department of Electrical and Computer Engineering at Boston University, MA, USA

***Biological processes***

**Table A. Growth and decay rates for HET, AOB, NOB, EPS and dead cells.** Here $\mu_{m,i}$and $b_{i}$are the maximum specific growth rate and decay constant for biomass group *i* (*i*=HET, AOB, NOB, EPS, I); $\eta_{H}$ is the reduction factor in anoxic conditions; $K_{j,i}$is the affinity constant between nutrient *j* and biomass group *i*; $S_{j}$is the concentration of nutrient *j* (*j*=S, NH_4_, O_2_, NO_2_, NO_3_).

| **Process** | **Rate (1/s)** |  |
| --- | --- | --- |
| Aerobic growth of HET | $\mu_{m,HET}\frac{S_{S}}{K_{S,HET}+S_{S}}\frac{S_{O2}}{K_{O2,HET}+S_{O2}}$ | R_1_ |
| Aerobic growth of AOB | $\mu_{m,AOB}\frac{S_{NH4}}{K_{NH4,AOB}+S_{NH4}}\frac{S_{O2}}{K_{O2,AOB}+S_{O2}}$ | R_2_ |
| Aerobic growth of NOB | $\mu_{m,NOB}\frac{S_{NO2}}{K_{NO2,NOB}+S_{NO2}}\frac{S_{O2}}{K_{O2,NOB}+S_{O2}}$ | R_3_ |
| Anoxic growth of HET on NO_3_ | $\eta_{H}\mu_{m,HET}\frac{S_{S}}{K_{S,HET}+S_{S}}\frac{S_{NO3}}{K_{NO3,HET}+S_{NO3}}\frac{K_{O2,HET}}{K_{O2,HET}+S_{O2}}$ | R_4_ |
| Anoxic growth of HET on NO_2_ | $\eta_{H}\mu_{m,HET}\frac{S_{S}}{K_{S,HET}+S_{S}}\frac{S_{NO2}}{K_{NO2,HET}+S_{NO2}}\frac{K_{O2,HET}}{K_{O2,HET}+S_{O2}}$ | R_5_ |
| Decay of HET | $b_{HET}$ | R_6_ |
| Decay of AOB | $b_{AOB}$ | R_7_ |
| Decay of NOB | $b_{NOB}$ | R_8_ |
| Decay of EPS | $b_{EPS}$ | R_9_ |
| Decay of Dead | $b_{I}$ | R_10_ |

**I Agent growth/decay**

The growth of particulate components (HET, AOB, NOB, EPS, I) is calculated using the following growth kinetic equation.

(1)

Here, *m*_i_ is the mass and *r_i_* is the specific growth/decay rate of the particulate component *i*. The specific growth/decay rates for various processes are listed in Table A.

According to Table A, the total growth/decay rates for each particulate component are calculated as below:

$$\begin{matrix} \frac{dm_{HET}}{dt}=\left[(R_{1}+R_{4}+R_{5})-(R_{_{6}})\right]m_{HET} \\ \frac{dm_{AOB}}{dt}=\left[(R_{2})-(R_{7})\right]m_{AOB} \\ \frac{dm_{NOB}}{dt}=\left[(R_{3})-(R_{8})\right]m_{NOB} \\ \frac{dm_{EPS\_H}}{dt}=\frac{Y_{EPS}}{Y_{HET}}(R_{1}+R_{4}+R_{5})m{}_{HET} \\ \frac{dm_{EPS}}{dt}=-R_{9}m_{EPS} \\ \frac{dm_{I}}{dt}=-R_{10}m_{I} \end{matrix}$$

When calculating above growth/decay rates, the nutrient concentrations (S_S_, S_NH4_, S_NO2_, S_NO3_, S_O2_) appeared in the expressions are the nutrient concentration at the voxel (Cartesian grid element) where the particulate component resides.

The above growth kinetic equation is discretized using an Euler explicit scheme as below:

$m_{i}(t+\Delta t_{bio})=m_{i}(t)+\Delta t_{bio}.m_{i}(t)*r_{i};i=HET,AOB,NOB, EPS\_H, EPS, I$,

where, $\Delta t_{bio}$ is the biological time step (it could be in the order of hours), *mEPS_H* and *m_EPS_* are EPS bound to HET and EPS particles, respectively. *Y_EPS_* and *Y_HET_* are yield coefficients explained below.

**II Nutrient uptake rates**

The stoichiometric matrix for particulate and soluble components is shown in Table B. The nutrient uptake rates for each soluble component at each voxel can be calculated by using Tables A and B as given below.

Here, *X_HET_*, *X_AOB_*, *X_NOB_*, *X_EPS_*, *X_I_* are concentrations of HET, AOB, NOB, EPS, and I respectively, at the voxel where the uptake rate is calculated, and they are calculated at voxel (*i*, *j*, *k*) as below.

$X_{HET}(i,j,k)=\frac{1}{\Delta x.\Delta y.\Delta z}\sum_{p=1}^{N_{HET}} m_{HET,p}$

$$X_{AOB}(i,j,k)=\frac{1}{\Delta x.\Delta y.\Delta z}\sum_{p=1}^{N_{AOB}} m_{AOB,p}$$

$$X_{NOB}(i,j,k)=\frac{1}{\Delta x.\Delta y.\Delta z}\sum_{p=1}^{N_{NOB}} m_{NOB,p}$$

$$X_{EPS}(i,j,k)=\frac{1}{\Delta x.\Delta y.\Delta z}\sum_{p=1}^{N_{EPS}} m_{EPS,p}$$

$$X_{I}(i,j,k)=\frac{1}{\Delta x.\Delta y.\Delta z}\sum_{p=1}^{N_{I}} m_{I,p}$$

Here, *N* is the number of respective agents within the voxel (Cartesian grid element), and ∆*x*, ∆*y*, ∆*z* are the dimensions of the voxel.

**Table B. Stoichiometric matrix for particulate and soluble components.** Here *Y_i_* is the yield coefficient for biomass group *i*. X and S are the particulate and soluble components, respectively. XEPS_H and X_EPS_ are the EPS associated with HET and EPS agents, respectively.

|  | **Particulate Components** | | | | | | **Soluble Components** | | | | |
| --- | --- | --- | --- | --- | --- | --- | --- | --- | --- | --- | --- |
|  | X_HET_ | X_AOB_ | X_NOB_ | XEPS_H | X_EPS_ | X_I_ | S_S_ | S_O2_ | S_NH4_ | S_NO2_ | S_NO3_ |
| Aerobic growth HET | 1 |  |  | $\frac{Y_{EPS}}{Y_{HET}}$ |  |  | $-\frac{1}{Y_{HET}}$ | $-\frac{1-Y_{HET}-Y_{EPS}}{Y_{HET}}$ |  |  |  |
| Aerobic growth AOB |  | 1 |  |  |  |  |  | $-\frac{3.42-Y_{AOB}}{Y_{AOB}}$ | $-\frac{1}{Y_{AOB}}$ | $\frac{1}{Y_{AOB}}$ |  |
| Aerobic growth NOB |  |  | 1 |  |  |  |  | $-\frac{1.15-Y_{NOB}}{Y_{NOB}}$ |  | $-\frac{1}{Y_{NOB}}$ | $\frac{1}{Y_{NOB}}$ |
| Anoxic growth HET on NO_3_ | 1 |  |  | $\frac{Y_{EPS}}{Y_{HET}}$ |  |  | $-\frac{1}{Y_{HET}}$ |  |  |  | $-\frac{1-Y_{HET}-Y_{EPS}}{2.86Y_{HET}}$ |
| Anoxic growth HET on NO_2_ | 1 |  |  | $\frac{Y_{EPS}}{Y_{HET}}$ |  |  | $-\frac{1}{Y_{HET}}$ |  |  | $-\frac{1-Y_{HET}-Y_{EPS}}{1.71Y_{HET}}$ |  |
| Decay HET | -1 |  |  |  |  |  | 1 |  |  |  |  |
| Decay AOB |  | -1 |  |  |  |  | 1 |  |  |  |  |
| Decay NOB |  |  | -1 |  |  |  | 1 |  |  |  |  |
| Decay EPS |  |  |  |  | -1 |  | 1 |  |  |  |  |
| Decay  Dead |  |  |  |  |  | -1 | 1 |  |  |  |  |

**III Agent division**

If the mass of the agent (m) reaches a certain threshold value, it divides into two daughter agents as described in the main text.

***Physical processes***

**I Nutrient mass balance**

Nutrient distribution within the rectangular computational domain is calculated by solving advection-diffusion-reaction equation (transport equation) for each nutrient (*S*_S_, *S*_NH4_, *S*_NO2_, *S*_NO3_, *S*_O2_). For any nutrient *S*, the mass balance is given by,

$\frac{\partial S}{\partial t}+\vec{U}\cdot\nabla S=\nabla\cdot(D_{e}\nabla S)+R$ (2)

The nutrient uptake rate *R* is calculated in Section (II) above.

Computational domain

|  |  |  |  |
| --- | --- | --- | --- |
|  |  |  |  |
|  |  |  |  |
|  |  |  |  |

i , j, k

z

y

*U_z_*

*U_x_*

*U_x_*

*U_z_*

Microbes

x

i,j,k

i+1/2,j,k

i-1/2,j,k

i,j+1/2,k

i,j-1/2,k

x

y

Cubical grid cell

**Fig A. Marker-And-Cell (MAC) scheme.**

The transport equation is discretized on a Marker-And -Cell (MAC) uniform grid shown in Fig A. The scalar *S* is defined at the centres of the voxel (cubical grid element) and the velocity components *U*=(*U_x_*, *U_y_*, *U_z_*) are defined at the centres of six faces of the voxel. The temporal derivative and spatial derivatives of the transport equation are discretized by Forward Euler and Central Finite Differences, respectively. For a given nutrient concentration field at time *t*, the concentration field at next time step can be calculated using following discretized equations.

When solving this equation, the effective diffusion coefficient *D_e_* is considered as a variable and it is calculated using local biomass information as seen in [1]. For example, at point (*x*,*y*,*z*) the effective diffusion coefficient of nutrient *S* is given by

$$D_{e}(x,y,z)=\left[1-\frac{0.43X^{0.92}(x,y,z)}{11.19+0.27X^{0.99}(x,y,z)}\right]D_{s}(x,y,z)$$

where *D_s_* (*x*,*y*,*z*) and *X* (*x*,*y*,*z*) are the diffusion coefficient of *S* in water and the total biomass concentration at point (*x*,*y*,*z*).

**II Mechanical relaxation**

The equations for the mechanical equilibrium of each particle is given by:

(3)

The forces on the right-hand side of the equation are due to contact (*F_c_*), fluid flow (*F_f_*), and adhesion (*F_a_*). These are calculated as described in the main text (Method section).

***Non-dimensionalization***

For simplicity, we rewrite equations (1-2) for a single species and single nutrient (carbon substrate) biofilm growing in a quiescent environment and we assume that the diffusion coefficient is constant.

$\frac{dm}{dt}=\left(\mu_{m}\frac{S_{S}}{K_{S}+S_{S}}-b\right)m$ (4)

$\frac{\partial S{}_{S}}{\partial t}=D_{S}\nabla^{2}S_{S}-\frac{\mu_{m}}{Y}\frac{S_{S}}{K_{S}+S_{S}}X$ (5)

The reference parameters for the non-dimensionalization are chosen as below:

Length=height of the simulation box, *L_z_*

Density=biomass density, *ρ_X_*

Nutrient concentration=Bulk nutrient concentration, *S_b_*

Time=1/growth rate=1/*µ_m_*

Then the equations (4-5) can be written in the non-dimensional form as

$\frac{dm^{*}}{dt^{*}}=\left(\frac{S^{*}}{\kappa+S^{*}}-\beta\right)m^{*}$ (6)

$0=\delta^{2}\nabla^{2}S^{*}-\frac{S^{*}}{\kappa+S^{*}}X^{*} (for steady state)$ (7)

The non-dimensional constants$\delta$, $\kappa$, and $\beta$ are given as

$$\beta=\frac{b}{\mu_{m}}$$

$$\delta=\sqrt{\frac{S_{b}D_{S}Y}{\mu_{m}\rho_{X}L_{z}^{2}}}$$

$\kappa=\frac{K_{S}}{S_{b}}$**.**

Here $m^{*},S^{*},X^{*},t^{*}$are the non-dimensional values of bacterial mass, nutrient concentration, biomass concentration, and time, respectively.

***Pseudocode of the model***

The pseudocode for the calculation procedure of the present model is shown below.

1. Update growth rates from local nutrient concentrations
2. Update biomass using a large time step of *O*(1000 s)
3. Update local nutrient concentrations using a very small time step of *O*(0.0001 s)
4. If EPS shell around HET >threshold value for EPS excretion, then create a new EPS particle
5. If active agent size >threshold value for division, then divide into two daughter agents
6. Mechanical Relaxation to calculate the equilibrium configuration. The equilibrium is decided by when the internal pressure of the system relaxes. This calculation is done at a time step around *O*(0.001 s).
7. Go to step (i).

***Model implementation in LAMMPS***

The classical LAMMPS provides different particle styles (or “atom styles” as commonly called in LAMMPS) that could be used in the simulation (<http://lammps.sandia.gov/doc/Manual.html>). The choice of particle style depends on what attributes are associated with the particles. In this IbM implementation in LAMMPS, a new particle style is implemented (named “bio”) for this IbM model. The new style is inherited from already existing atom style sphere in the granular package which has default attributes such as mass, diameter and velocity. In addition to the default attributes, each “bio” style atom has following two attributes in conjunction to the sphere type: outer-diameter and outer-density to incorporate EPS shell around HET particles. For AOB and NOB both outer and inner radii are the same since they do not produce EPS in this model.

In LAMMPS, “fix” is any operation that applies to the system during time integration. Example includes updating of particle locations, velocities, forces etc. These fixes together with their related parameters are listed in the input script. The present IbM implementation in LAMMPS defines a series of new fixes to perform the IbM related operations mentioned in the Method section of the main text. These new operations include particle growth, division, EPS production, death, nutrient transport etc. During the simulation, fixes are invoked in a user-defined frequency to update particle attributes. Examples include calculating the forces applied on microbes, updating the positions and velocities due to time integration, calculating growth and death, etc. After particle growth, the time integration to update the locations of overlapping partciles (i.e., solving Equation 3) is done using the Velocity-Verlet integrator which is the default in LAMMPS. This time integration is done using one of the LAMMPS inbuilt integrator “fix nve/limit”. During this time integration a limit is imposed on the maximum distance a particle can move in one time step. This limit avoids particles blowing due to highly overlap particles at the start of the integration after each growth step. The limit we used was 1x10^-8^µm or less.

Detailed descriptions of all the fixes and how to use them are detailed in the documentation of the model. The documentation and source code of the model, and some helpful examples are given at <https://github.com/nufeb/NUFEB>. The results shown in this paper can be reproduced by running this code with the relevant parameters in the input script given in the repository.

***Mechanical equilibrium of biofilm***

In this model, the mechanical equilibrium is obtained when the average pressure of the biofilm/floc releases and reaches the equilibrium state. The average pressure of the system due to inter-particle interactions are calculated as [2,3]

(8)

where, $\vec{r}_{ij}$ and $\vec{F}_{ij}$are the distance and force between two interacting particles *i* and *j*. $m_{i}$and $\vec{v}_{i}$ are the mass and translational velocity of particle *i*. The first and second term in the bracket shows the contributions from kinetic and interaction energy, respectively. LAMMPS [2] defines *V* as the volume of the computational domain, but in this work, *V* is redefined as the sum of the volumes of individual particles since the pressure is then averaged over the biofilm volume rather than whole computational domain.

The biofilm shown in Fig 3 of the main text is grown for 40 hours without any mechanical relaxation and then the internal pressure is released by solving equation 3. Fig B shows how the internal pressure of a biofilm decreases over the time the mechanical relaxation is performed. It is seen that the pressure rapidly decreases and approaches its equilibrium value within one to two seconds. There would be a very small amount of residual stress at the equilibrium configuration (in this case it is about 4.38 Pa, which is very small compared to the initial pressure), but the shape or positions of the particles would not change significantly at this residual pressure.


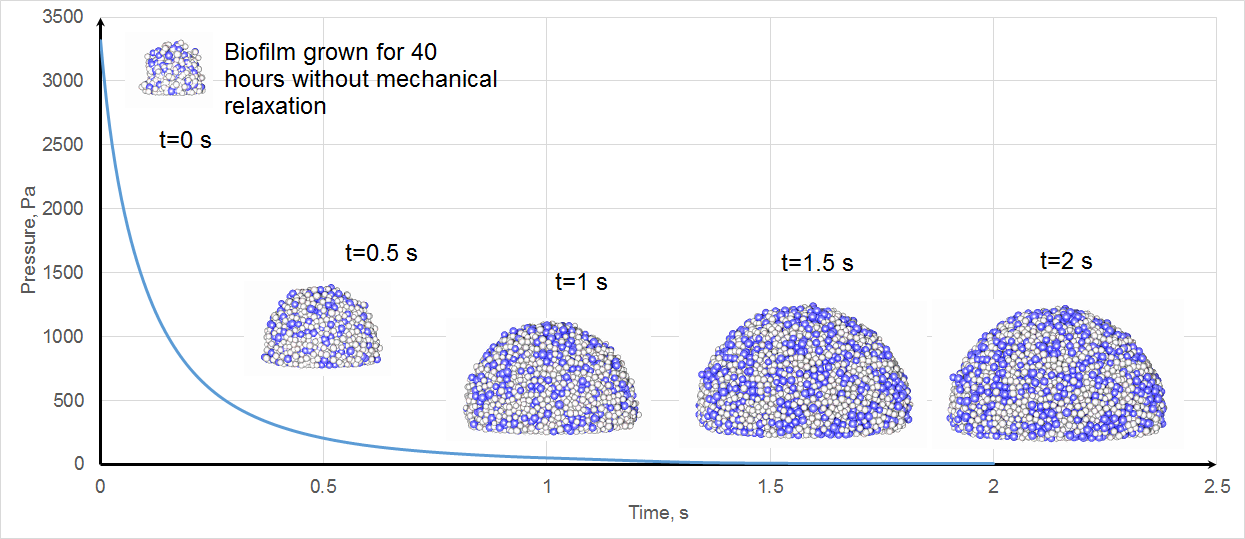


**Fig B. Mechanical relaxation of a biofilm.** The biofilm is grown for 40 hours as in Fig 3, but without mechanical relaxation, and then the relaxation is done for 2 s so that it is clear to visualize how the shape changes as the internal pressure releases and finally it approaches the equilibrium configuration.


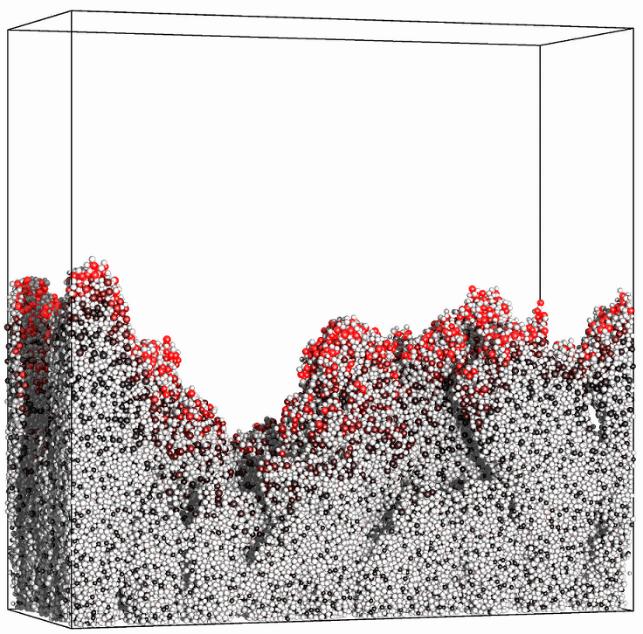

**Fig C. Active layer of biofilm for very low nutrient conditions** ($\delta$=1.52×10^-2^ and $\kappa$=0.35, the left-bottom point of the plots shown in Figs 5-6)**:** (a) the growth rates of bacterial cells after 13 days of growth. The colour of bacterial cells varies black to red with proportional to the growth rate (highest growth rate is shown red). The gray colour particles are EPS. It is seen that only the bacterial cells at the top of the biofilm are active; (b) variation of average active layer thickness over time. The active layer thickness at any point on the surface is calculated as$\frac{(dm/dt)/m}{-\frac{d[(dm/dt)/m]}{dz}}$ , where *m* is the mass of the agent residing on the surface. This expression simply calculates at which distance from the surface the growth rate of agents becomes zero. The average active layer thickness is the average of above expressions calculated at all the voxels on the biofilm surface. More details about this calculation is seen in [4].

(b)

(a)

Shear rate = 0.12 s^-1^


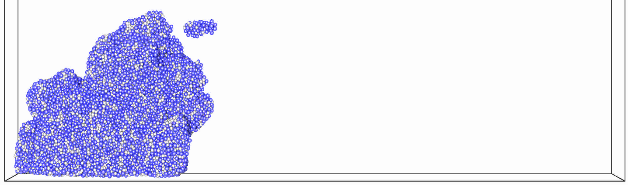

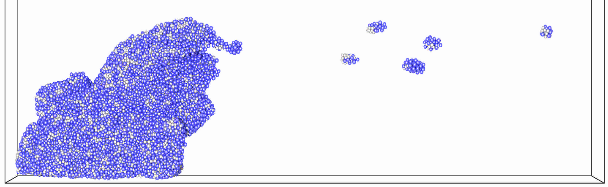


Shear rate = 0.16 s^-1^


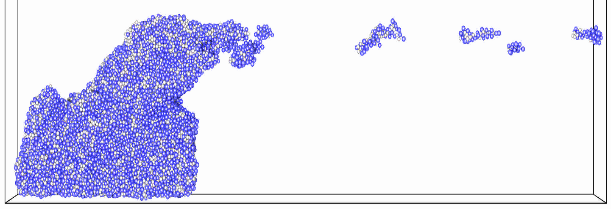

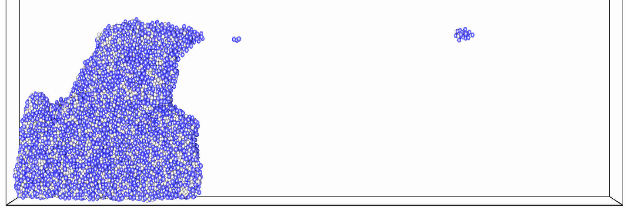


Shear rate = 0.20 s^-1^


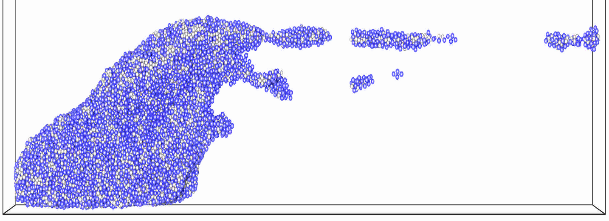

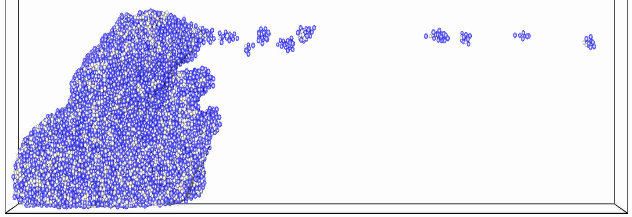


Shear rate = 0.24 s^-1^


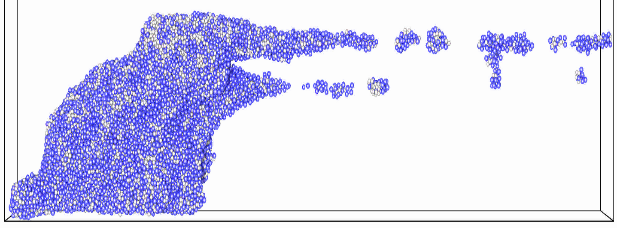

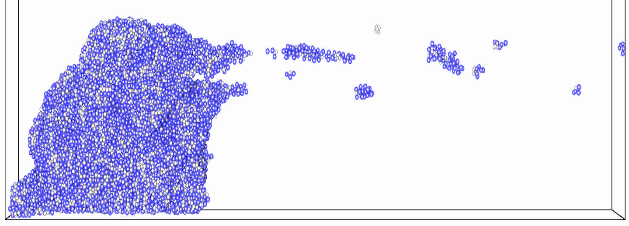


**Fig D. Biofilm deformation and detachment at different shear rates.** The left column is at T^**^=20000 and the right column is at T^**^=70000. The results indicate that higher shear rates would result in elongated steamers.

**References**

1. Fan LS, Leyvaramos R, Wisecarver KD, Zehner BJ (1990) Diffusion of Phenol through a Biofilm Grown on Activated Carbon Particles in a Draft-Tube 3-Phase Fluidized-Bed Bioreactor. Biotechnology and Bioengineering 35: 279-286.

2. LAMMPS LAMMPS manual, <http://lammps.sandia.gov/doc/Manual.html>.

3. Heinz H, Paul W, Binder K (2005) Calculation of local pressure tensors in systems with many-body interactions. Physical Review E 72.

4. Head DA. Linear surface roughness growth and flow smoothening in a three-dimensional biofilm model. Phys Rev E. 2013;88(3). doi: ARTN 032702

10.1103/PhysRevE.88.032702. PubMed PMID: WOS:000323895200006.
